# Prevalence of overweight and obesity in incarcerated individuals in developed and developing countries: A systematic review and meta‐analysis

**DOI:** 10.1111/obr.13906

**Published:** 2025-02-12

**Authors:** Leonida Nyarwaba Mosomi, Magaly Aceves‐Martins, Alexandra M. Johnstone, Baukje de Roos

**Affiliations:** ^1^ The Rowett Institute University of Aberdeen Aberdeen UK

**Keywords:** diet, incarcerated individuals, obesity, overweight

## Abstract

We systematically assessed evidence of overweight and obesity prevalence, and possible determinants, in people who experience incarceration globally. We searched Embase, Medline, and Cochrane databases. Overweight and obesity proportions were pooled into a meta‐analysis and compared with national prevalences.

Seventy‐one studies met the inclusion criteria; 38 were included in the meta‐analysis. Studies in high‐income countries reported higher proportions of overweight and obesity (73.3%, 95% CI 73.1, 73.5) than upper‐middle‐income countries (66.1%, 95% CI 64.1, 67.7) and lower‐middle‐income countries (52.8%, 95% CI 47.1, 58.1). The prevalence of overweight and obesity in female incarcerated individuals was higher than that in the general population (RD 11.7%, 95% CI 9.1, 14.3), especially in low and middle‐income countries (RD 35.1%, 95% CI 29.4, 40.7). The prevalence of overweight and obesity in male incarcerated individuals was lower than that in the general population in all income categories (RD ‐10.8%, 95% CI ‐13.2, −8.4). Incarcerated individuals in low and lower‐middle‐income countries were less likely to achieve sufficient energy intake, fruit/vegetable intake, and sufficient physical activity, compared with high‐ and upper‐middle‐income countries.

The prevalence of overweight and obesity in incarcerated populations differed between developed and developing countries. Future research should focus on female incarcerated individuals, especially in lower‐middle‐income countries.

## 1 INTRODUCTION

People who experience incarceration are vulnerable institutionalized populations whose daily lifestyle and health status can be affected by incarceration. In 2021, the Institute for Criminal Policy Research reported that incarcerated populations are increasing, with the current total incarcerated population estimated to be around 10 million worldwide.^1^ The dietary intake and physical activity of incarcerated individuals are limited to what their national or institutional policies offer or allow.^2^, ^3^ Although most countries aim to ensure that incarcerated individuals have access to healthcare,^2^, ^4^ constrained resources and overpopulated carceral facilities often compromise carceral policies, especially in developing countries that are faced with both infectious diseases and non‐communicable diseases burden.^5^, ^6^, ^7^ As a result, incarcerated individuals often face more health challenges than the general population.^8^, ^9^

In developed countries, incarcerated individuals are at a greater risk of being overweight and obesity,^8^, ^10^ sedentary lifestyles,^11^, ^12^ and chronic illnesses,^13^, ^14^ with younger incarcerated individuals having a higher risk of cardiometabolic disease than the general population.^15^, ^16^ Also, incarcerated individuals tend to consume excess calories^17^ and more ultra‐processed foods,^18^, ^19^ making the overall diet nutritionally inadequate. Moreover, global reports suggest that developing countries currently face a double burden of malnutrition, with undernutrition and overnutrition happening simultaneously, including a rapid rise in the prevalence of overweight and obesity.^20^

Systematic reviews conclude that excess weight gain is a major risk factor for non‐communicable diseases in incarcerated individuals, with weight gain being influenced by a sedentary lifestyle, intake of an unhealthy diet, stress, and substance abuse among incarcerated individuals.^8^, ^9^, ^21^ In addition, excess weight may increase the risk of metabolic disorders,^22^, ^23^ affecting the overall health status and increasing the financial burden on medical care within prisons and the community once incarcerated individuals are released after incarceration.^24^ Therefore, improving diet quality should be considered a health promotion priority within carceral facilities.^25^

There are limited systematic reviews evaluating the prevalence of overweight and obesity in incarcerated individuals and the differences in prevalence that might exist between developed and developing countries.^8^, ^9^, ^21^ For this reason, the present systematic review aims to assess the prevalence of overweight and obesity of incarcerated populations in developing and developed countries, as well as possible determinants of overweight and obesity, including dietary intake and physical activity.

## 2 METHODS

This systematic review was registered with the International Prospective Register of Systematic (PROSPERO registration no CRD42022320861) and is reported according to Preferred Reporting Items for Systematic Reviews and Meta‐analyses (PRISMA) guidelines.

### 2.1 Selection criteria

The inclusion criteria of the Population, Exposure, Comparator, Outcomes, and Study (PECOS) framework are described in Table 1.

**TABLE 1:** Eligibility criteria according to the PECOS framework.

	Criteria
**Population**	Studies considering healthy male or female adults (≥18 years old). Studies assessing war prisoners, ex‐prisoners (no longer incarcerated), juvenile prisoners (under 18 years of age), or prisoners with chronic diseases were excluded.
**Exposure**	Study populations had to be incarcerated (serving a sentence in prison) at the time of the study.
**Comparator**	Any or none. Studies with or without a control group were considered.
**Outcome**	Primary outcomes included anthropometric measurements (including body weight, body height, and/or BMI), and the prevalence of overweight or obesity. Secondary outcomes included dietary intake (patterns, macronutrient intake, and/or food composition), micronutrient levels, or physical activity levels.
**Study design**	Cross‐sectional studies or baseline data from longitudinal or intervention studies.

### 2.2 Search strategy

Searches were done on Medline, Embase, and Cochrane databases. A sensitive search strategy based on the PECOS criteria was designed using a combination of words, synonyms, and Boolean connectors, such as “prisoners”, “prisons”, “nutrition”, and “nutritional status”, to retrieve relevant studies. References of included studies were hand‐searched to identify other potentially eligible studies. The complete search strategy is available in Table S1.

### 2.3 Data selection and extraction

Titles and abstract screening was conducted 100% by one reviewer (LM) and cross‐checked at 10% for validation purposes by a second reviewer (MA‐M). Full‐text screening and data extraction were conducted by one reviewer (LM) and checked by a second reviewer (MA‐M). A data extraction form was created based on the PECOS criteria. Data extracted included general information on the study (publication year, study design); setting (country‐categorized by region and by income,^26^ levels of prison security [maximum, medium, minimum]); population (age, sex, duration of incarceration, participants data); anthropometric indicators (weight, height, BMI, reference used to categorize BMI, how weight and height were measured); overall primary outcomes, and secondary outcomes (dietary intake, markers of metabolic syndrome, physical activity, and micronutrient deficiencies).

### 2.4 Synthesis and statistical analysis

The countries where the studies were executed were categorized according to World Bank 2023 fiscal year income,^26^ measured using gross national income (GNI) per capita method in 2021, in US dollars, converted from local currency using the World Bank Atlas. Such categories included four income groupings: low (GNI of $1085 or less), lower‐middle (GNI of between $1086 and $4255), upper‐middle (GNI of $4256 and $13,205), and high (GNI of $13,205 or more). The main population characteristics and primary outcomes (i.e., body weight‐related outcomes) reported across studies were tabulated and synthesized narratively. Furthermore, dietary intake data were compared with the recommended dietary intake for energy and fruit and vegetable intake for each country.^27^, ^28^, ^29^, ^30^ Physical activity data were compared with the World Health Organization's recommendation on physical activity.^31^

For those studies providing sufficient data on the exact proportion of incarcerated individuals presenting overweight or obesity, a proportional meta‐analysis within the incarcerated population was conducted and compared with the national prevalences. First, the proportion of overweight and obesity within prisons in each study sample and the 95% confidence intervals (CI) were estimated by sex and stratified according to the income categories in each country. Additionally, national overweight and obesity prevalence data were retrieved from the World Obesity Federation Global Obesity Observatory^32^ for each country and adjusted for sex and year of data collection of the studies included in the meta‐analyses. The proportion of incarcerated individuals with overweight and obesity was then compared with that of the general population using national data through a risk difference (RD) meta‐analysis. The heterogeneity of studies included in the meta‐analyses was assessed through I^2^, and a random or fixed‐effect model was fitted accordingly. Data are presented graphically in forest plots. Analysis was done in R software using the library “*meta*” for data analysis and visualization.

### 2.5 Quality of studies critical appraisal

We used the JBI (formerly Joanna Briggs Institute) Critical Appraisal Checklist for Analytical Cross‐Sectional Studies to evaluate the risk of bias in individual cross‐sectional studies. The overall quality of the study was determined using a score for each criterion based on whether the studies considered each criteria (yes = 1, unclear = 0, and no = 0). To grade the studies, total scores were interpreted as follows: ≤4 poor quality, 5–6 moderate quality, and ≥7 good quality.

## 3 RESULTS

**FIGURE 1:**
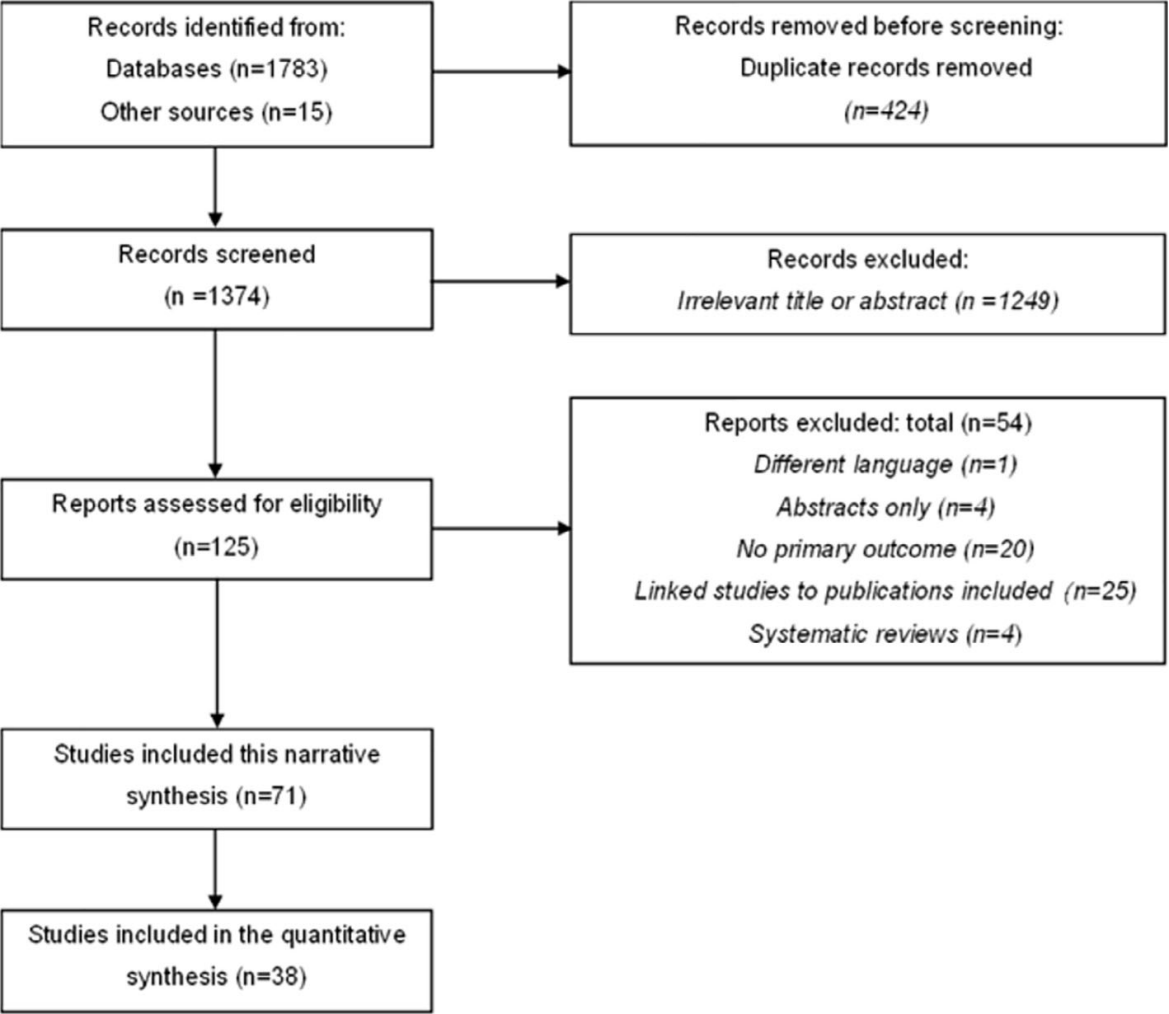
PRISMA flowchart.

**FIGURE 2:**
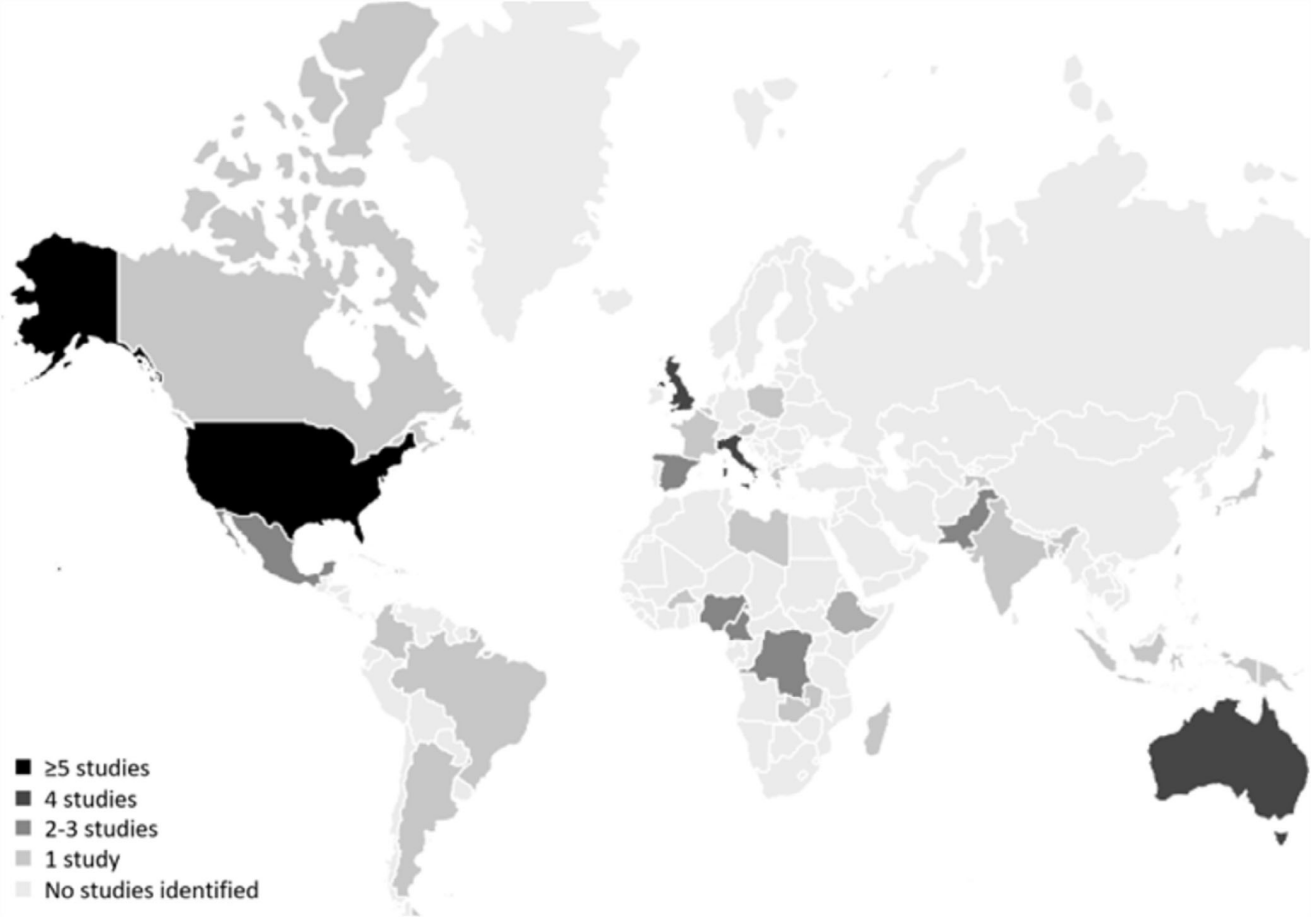
Geographical map of the evidence.

Based on the search strategy, 1374 references were identified, from which 125 were retrieved for full‐text review. Overall, 71 studies, reported in 75 references,^6^, ^10^, ^11^, ^12^, ^13^, ^15^, ^16^, ^17^, ^18^, ^19^, ^25^, ^33^, ^34^, ^35^, ^36^, ^37^, ^38^, ^39^, ^40^, ^41^, ^42^, ^43^, ^44^, ^45^, ^46^, ^47^, ^48^, ^49^, ^50^, ^51^, ^52^, ^53^, ^54^, ^55^, ^56^, ^57^, ^58^, ^59^, ^60^, ^61^, ^62^, ^63^, ^64^, ^65^, ^66^, ^67^, ^68^, ^69^, ^70^, ^71^, ^72^, ^73^, ^74^, ^75^, ^76^, ^77^, ^78^, ^79^, ^80^, ^81^, ^82^, ^83^, ^84^, ^85^, ^86^, ^87^, ^88^, ^89^, ^90^, ^91^, ^92^, ^93^, ^94^, ^95^, ^96^ met the inclusion criteria of the current review (Figure 1). Most studies (n = 68) had a cross‐sectional design, while three studies^11^, ^34^, ^75^ had a cohort design. Only baseline data from these three studies were included in this review. The publication years spanned from 1985^40^ ^4^ to 2022,^68^ and the studies were conducted across 34 countries (Figure 2). According to the World Bank Country classification,^26^ 43 studies were from high‐income countries,^10^, ^11^, ^12^, ^13^, ^15^, ^16^, ^17^, ^19^, ^25^, ^36^, ^38^, ^40^, ^44^, ^45^, ^49^, ^50^, ^51^, ^52^, ^53^, ^54^, ^55^, ^57^, ^58^, ^59^, ^61^, ^63^, ^64^, ^66^, ^68^, ^71^, ^72^, ^73^, ^75^, ^78^, ^82^, ^84^, ^85^, ^86^, ^87^, ^89^, ^90^, ^91^, ^92^, ^93^, ^95^, ^96^ 6 from upper‐middle‐income countries,^18^, ^34^, ^39^, ^48^, ^69^, ^76^ 16 from lower‐middle‐income countries,^33^, ^37^, ^42^, ^43^, ^47^, ^60^, ^62^, ^67^, ^70^, ^74^, ^77^, ^79^, ^80^, ^83^, ^88^, ^94^ and 6 from low‐income countries.^35^, ^41^, ^46^, ^56^, ^65^, ^81^ A total of 2,322,369 incarcerated individuals were studied in the included studies; 2.118,211 were male incarcerated individuals, and 204,158 were female incarcerated individuals (Table 2, ^6^, ^10^, ^11^, ^12^, ^13^, ^15^, ^16^, ^17^, ^18^, ^19^, ^25^, ^33^, ^34^, ^35^, ^36^, ^37^, ^38^, ^39^, ^40^, ^41^, ^42^, ^43^, ^44^, ^45^, ^46^, ^47^, ^48^, ^49^, ^50^, ^51^, ^52^, ^53^, ^54^, ^55^, ^56^, ^57^, ^58^, ^59^, ^60^, ^61^, ^62^, ^63^, ^64^, ^65^, ^66^, ^67^, ^68^, ^69^, ^70^, ^71^, ^72^, ^73^, ^74^, ^75^, ^76^, ^77^, ^78^, ^79^, ^80^, ^81^, ^82^, ^83^, ^84^, ^85^, ^86^, ^87^, ^88^, ^89^, ^90^, ^91^, ^92^, ^93^, ^94^, ^95^, ^96^). The study sample sizes varied from 30^76^ to 1,441,800^36,49^ incarcerated individuals. Seven studies were conducted in minimum security prisons, accommodating individuals incarcerated for the first time, non‐violent incarcerated individuals, and those on parole or due for release who may work outside the prison camps. Fifteen studies were conducted in medium security prisons, 16 in maximum security prisons, 28 in mixed‐level prisons, and four studies^45^, ^69^, ^75^, ^93^ did not report the level of prison security.

**TABLE 2:** Summary of study characteristics, findings, and quality.

STUDY ID Country Data collection	Sample Sample size (n) Male (%) Female (%)	BMI categorization and reference methodology	Overweight and obesity combined prevalence (%)	Reported outcomes	Quality critical Appraisal^a^
**High‐Income Countries**					
**Al‐Rousan 2017** ^10^ United States 2015	n = 8574 M = 91% F = 9%	WHO reference. Weight/height measured	77.7%	40.1% of incarcerated individuals had overweight, 37.6% had obesity and 0.3% had underweight. No dietary or PA outcomes were reported in the study.	Good (7/8)
**Johnson 2018** ^11^, ^64^ Canada 2016–2017	n = 754 M = 89% F = 11%	WHO reference. Weight/height measured	81.8%	39.4% of the incarcerated individuals had overweight, 42.4% had obesity, and no incarcerated individuals had underweight. 30% reported eating <1 vegetable serving per day, while 25% reported <1 fruit a day. Incarcerated individuals who gained the most weight (15.7 kg) reported not eating vegetables, whereas incarcerated individuals who gained less weight (4.8 kg) reported consuming vegetables over three times per day. Most incarcerated individuals (95%) purchased food from the commissary store or canteen. Younger incarcerated individuals typically reported making healthier food choices from the commissary store or canteen than older incarcerated individuals; weight gain was less severe for incarcerated individuals who engaged in regular PA, 42% performing <30 minutes of PA.	Good (8/8)
**Lagarrigue 2017** ^12^ France 2012–2013	n = 51 M = 35% F = 65%	WHO reference. Weight/height measured	43.1%	30.3% of female incarcerated individuals and 11% of male incarcerated individuals had overweight. 21.2% of female incarcerated individuals and 16.7% male incarcerated individuals had obesity. 5.6% of female incarcerated individuals and 6.1% male incarcerated individuals had underweight. Authors reported that incarceration worsened the rate of obesity in males and females. Abdominal obesity (waist circumference) was prevalent in women (69.7%) and men (27.8%). The prison provided meals, but there were no differences between sexes, both were served a diet of ~2400 kcal/day. Incarcerated individuals were reported to eat in their cells and were able to purchase food at the prison store. More females than males were reported as inactive (37.9% vs 11.8%), and fewer were reported as very active (17.2% vs 41.2%).	Good (7/8)
**Gates 2015** ^13^ United States 2005–2011	n = 2932 M = 93% F = 7%	CDC reference. Weight/height measured	NR (only mean BMI presented)	Both male and female incarcerated individuals gained weight during incarceration; female incarcerated individuals gained significantly more weight than males. However, incarcerated individuals with diabetes (ΔBMI = −0.39) and hypertension (ΔBMI = 0.06) did not gain more weight than incarcerated individuals who did not have these chronic diseases. No dietary or PA outcomes were reported in the study.	Good (7/8)
**D'Souza 2005** ^15^ Australia 1996 and 2001	n = 1705 M = 82% F = 18%	WHO reference. Weight/height measured	49.1%	33.4% of the incarcerated individuals had overweight, 15.7% had obesity and no incarcerated individuals had underweight. There was a high prevalence of cardiovascular disease risk factors, particularly in younger incarcerated individuals, compared with the Australian non‐prison population, including high BMI.	Good (8/8)
**Gray 2021** ^16^ United Kingdom 2019	n = 299 M = 100% F = 0	WHO reference. Weight/height measured	80.9%	43.5% of the incarcerated individuals had overweight, 37.5% had obesity and no incarcerated individuals had underweight. 48.7% of the incarcerated individuals met PA guidelines. No dietary outcomes were presented in this study.	Good (7/8)
**Hannan‐Jones 2016** ^17^ Australia NR	n = 120 M = 100% F = 0	WHO reference. Weight/height measured	72.5%	58.3% of the incarcerated individuals had overweight, 14.2% had obesity and 0.8% had underweight. High energy intakes were reported with a median of 13.8 (SE 0·3) of self‐funded snacks. Sharing, trading up, and swapping of food were frequently reported among incarcerated individuals. Prevalence of diabetes and heart disease risk was similar to that in the general population. Prevalence of obesity was lower, and prevalence of smoking was higher than that in the general population. Self‐report of daily PA was 84%, with 51% taking part ≥2 times daily.	Good (7/8)
**Nucci 2020** ^19^ Italy 2017	n = 22 M = 50% F = 50%	WHO reference. Weight/height measured	52.0%	36% of incarcerated individuals had overweight, 16% had obesity and 1.2% had underweight. 45.3% of incarcerated individuals ate carbohydrates over four times per week compared to 34.9% who did so before imprisonment. Consumption of fruit and vegetable (over seven times/week) was reduced after imprisonment (41.9% before vs 37.2% after), as well as fish (from 1 to 7 times/week, 73.3% before vs 67.4% after); legumes (at least 4 times/week, 25.6% before vs 19.8% after); nuts (at least 4 times/week, 29.1% before vs 12.8% after) and whole grain foods (at least 4 times/week, 29.1% before vs 12.8% after). Low consumption of meat (only three times per week) was more common during imprisonment than before (36.1% before vs 62.8% after), as was low consumption of milk and dairy products (only seven times per week, 54.6% before vs 65.1% in prison). Sweets and soft drink consumption increased during detention (41.9% before vs 57.0% after and 43.0% before vs 55.8% after, respectively). 45.3% were physically active for at least 30 min twice a week.	Good (7/8)
**Plugge 2009** ^25^ United Kingdom 2004	n = 430 M = 0 F = 100%	WHO reference. Weight/height measured	29.5%	29.5% of incarcerated individuals had overweight or had obesity and 26.5% had a BMI of 20 or less. Only 12.7% met the national fruit and vegetable consumption recommendations and were more likely to drink semi‐skimmed or skimmed milk (p < 0.001). Women incarcerated individuals were at high risk of cardiovascular disease; 85% smoked cigarettes, 87% were insufficiently active to benefit their health, and 8% of women incarcerated individuals vs 27% of the general adult female population met these guidelines.	Good (7/8)
**Shaw 1985** ^40^ United States 1985	n = 56 M = 0 F = 100%	Metropolitan Height and weight tables Weight/height measured	48.5%	48.5% of incarcerated individuals had overweight or had obesity; no incarcerated individuals had underweight. Incarcerated individuals incurred significant weight gain as the average caloric intake exceeded 174% of the Recommended Daily Allowance (RDA). No data of PA were presented in this study.	Good (7/8)
**Maruschak 2015** ^36^, ^49^ United States 2011–2012	Prison incarcerated individuals n = 1,441,800 M = 93% F = 7% Jail incarcerated individuals n = 720,200 M = 87% F = 13%	WHO reference. Weight/height measured	Prison population 73.6% Jail population 61.6%	For male prison population, the BMI reports were as follows: 0.8% underweight, 25.8% normal weight, 46.5% overweight, 24.7% obesity, and 2.2% morbid obesity. For female prison population, the BMI reports were as follows: 1.0% underweight, 21.6% normal weight, 34.7% overweight, 37.2% obesity, and 5.6% morbid obesity. For male jail population, the BMI reports were as follows: 1.2% underweight, 38.3% normal weight, 40.3% overweight, 18.8% obesity, and 1.4% morbid obesity. For female jail population, the BMI reports were as follows: 1.8% underweight, 29.7% normal weight, 31.8% overweight, 28.6% obesity, and 8.0% morbid obesity. Prison population with overweight and obesity had lower rates of inmate‐on‐inmate sexual victimization and staff misconduct than population with or below a normal weight (p < 0.05). In addition, inmate‐on‐inmate sexual victimization rates among prison population were higher among females (6.9%) than males (1.7%). No data of dietary or PA were reported in the study.	Good (7/8)
**Rocca 2018** ^38^ Italy 2015	n = 142 M = 100% F = 0	CDC reference. Weight/height measured	66.9%	45.8% of incarcerated individuals had overweight, 21.1% had obesity, and no incarcerated individuals had underweight. The prevalence of obesity and overweight among incarcerated individuals (66.9%) was higher than the general Italian population (54.8%, adult males, ISTAT, 2015). No dietary or PA outcomes were reported in the study**.**	Good (7/8)
**Clarke 2012** ^44^ United States 2007–2008	n = 152 M = 0 F = 100%	National Institutes of Health, 1998 reference. Weight/height measured	67.0%	34.9% of the incarcerated individuals had overweight, 32.1% had obesity and no incarcerated individuals had underweight. The obesity prevalence was higher than among women in the community in Rhode Island (32% vs 21%). Most study participants were reported to gain weight during incarceration. Women gained an average of 0.5kgs per week, and the average weekly weight gain was higher among women, more recent incarcerated individuals. Incarcerated individuals were allowed to order food from the prison store, mostly high‐calorie foods with little nutritional value. No data on PA was presented in this study.	Good (7/8)
**Lai 2008** ^45^ Taiwan 2004–2005	n = 1129 M = 100% F = 0	Department of Health Taiwan reference Weight/height measured	15.0%*	15% of the incarcerated individuals had obesity. No dietary or PA outcomes were reported in the study.	Good (7/8)
**Befus 2015** ^50^ United States 2011–2013	n = 1432 M = 55% F = 45%	WHO reference. Weight/height measured	74.8%	52.1% of the male incarcerated individuals and 32.4% of the female incarcerated individuals had overweight, and 26.2% of the male and 37.9% of the female incarcerated individuals had obesity. No incarcerated individuals had underweight. No dietary or PA outcomes were reported in the study.	Good (7/8)
**Binswanger 2009** ^51^ United States 2002–2004	Prison incarcerated individuals n = 14,373 M = 77% F = 23% Jail incarcerated individuals n = 6582 M = 70% F = 30%	WHO reference. Self‐reported weight/height measures	Prison population 67.7% Jail population 52.9%	In prisons, 43.9% of the incarcerated individuals had overweight, 23.8% had obesity and 0.4% had underweight. In jails, 36.1% of the incarcerated individuals had overweight, 16.8% had obesity and 1.8% had underweight Prison population had higher odds of obesity than jail population (jail OR 0.45; 95%CI 0.40–51), (OR prison 0.80; 95% CI 0.72 to 0.88). Although both had lower odds of obesity than non‐institutionalized adults. No dietary or PA outcomes were reported in the study.	Moderate (6/8)
**Brewer‐Smyth 2014** ^52^ United States NR	n = 81 M = 0 F = 10%0	CDC reference. Unclear how weight/height was measured	43.2%*	43% of the incarcerated individuals had obesity. Authors reported that obesity might result from painful previous experiences, including childhood (OR = 1.21; 95%CI 1.02–1.43) and physical or emotional trauma (OR = 1.01; 95%CI 0.94–1.08). Also, obesity was associated with traumatic brain injury (OR = 0.68; 95%CI 0.48–0.96) and self‐medication (OR = 0.26; 95%CI 0.06–1.19). No dietary or PA outcomes were reported in the study.	Moderate (5/6)
**Brewer‐Smyth 2016** ^53^ United States 2009–2010	n = 636 M = 50% F = 50%	CDC reference. Unclear how weight/height was measured	33.0%*	23.9% of male incarcerated individuals and 43.1% of female incarcerated individuals had obesity. Obesity was significantly more prevalent in females (OR = 2.41; 95%CI 1.71, 3.39) and older participants (OR = 1.04; 95%CI 1.02, 1.05). No dietary or PA outcomes were reported in the study.	Poor (4/8)
**Camplain 2021** ^54^ United States 2017–2018	n = 197 M = 79% F = 21%	WHO reference. Self‐reported weight/height measures	59.6%	36.8% of the incarcerated individuals had overweight, 22.8% had obesity and no incarcerated individuals had underweight. No dietary or PA outcomes were reported in the study.	Poor (4/8)
**Choudhry 2019** ^55^ United Kingdom NR	n = 367 M = 100% F = 0	WHO reference. Weight/height measured	16.0%*	16% of the incarcerated individuals had obesity. 84% were reported to meet recommended weekly PA levels (>150 minutes per week), and 74% consumed more than recommended daily calories for males (2500 kcal).	Moderate (5/8)
**Drach 2016** ^57^ United States 2014	n = 133 M = 0 F = 100%	CDC reference. Weight/height measured	87.0%	39% of the incarcerated individuals had overweight, 48% had obesity, and no incarcerated individuals had underweight. Authors report that the most used healthy strategies to lose or maintain weight included exercise (85%), drinking less soda or juice (85%), and eating fewer calories (84%) or less fat (81%). Women who used tobacco and illicit substances before incarceration gained more weight while incarcerated. 24% of the incarcerated individuals reported using one or more unhealthy strategies to lose weight in the past 6 months.	Good (7/8)
**Edwards 2001** ^58^, ^59^ United Kingdom 1999	n = 506 M = 100% F = 0	WHO reference. Self‐reported weight/height measures	NR (only mean BMI presented)	The mean BMI recorded per age group was: 21–30 years 23.74 (±3.11), 31–40 years 24.98 (± 3.79), 41–50 years 26.51 (± 4.65), 51–60 + years 26.24 (± 4.49). The mean nutrient intake from the food provided by the prison food service was 2561 kcals. Incarcerated individuals were not permitted to accept any food brought in by visitors, but they were reported to be able to purchase various items from the prison canteens. No data on PA outcomes was presented.	Moderate (6/8)
**Hachbardt 2020** ^61^ Italy 2016–2018	n = 157 M = 0 F = 100%	WHO reference. Weight/height measured	47.1%	24.2% of the incarcerated individuals had overweight, 22.9% had obesity and 3.8% had underweight. Participants had a low cardiovascular disease risk, but BMI values pointed to having overweight in all the years analyzed. Moreover, 91.5% of the incarcerated individuals in the study reported a sedentary/insufficiently active lifestyle. No dietary outcomes were presented in this study.	Good (7/8)
**Jacobs 2015** ^63^ United States NR	n = 59 M = 100% F = 0	WHO reference. Weight/height measured	66.1%	44.1% of the incarcerated individuals had overweight, 22% had obesity and no incarcerated individuals had underweight. 37.9% of incarcerated individuals serving short‐term and 90% of those serving long‐term sentences were vitamin D deficient. No dietary or PA outcomes were reported in the study.	Good (7/8)
**Khavjou 2007** ^66^ United States 2004–2005	n = 261 M = 0 F = 100%	WHO reference. Weight/height measured	71.0%	28.6% of the incarcerated individuals had overweight, 42.4% had obesity and no incarcerated individuals had underweight. The prevalence of overweight and obesity among incarcerated individuals vs non‐prisoner participants was significantly higher (71.0% vs 74.0%, p < 0.03). The prevalence was also higher among female incarcerated individuals (85%) than non‐imprisoned women (54%). No dietary or PA outcomes were reported in the study.	Good (7/8)
**Kosendiak 2022** ^68^ Poland 2020	n = 211 F = 100% M = 0	WHO reference. Weight/height measured	60.2%	41.7% of the incarcerated individuals had overweight, 18.5% had obesity and 0.9% had underweight. PA level was reported as follows: low level 44.8%, medium level 10.4%, and high level 45.5%. Incarcerated individuals aged 18–29 and over 40 declared having a low level of PA, whereas those aged 30–40 reported a high or medium level. The level of PA of the surveyed incarcerated individuals was not significantly correlated with their dietary habits, diet quality, or nutrition knowledge. 41.2% consumed three meals per day, 28.4% four meals per day, and 25.1% five meals daily. Bought food in the canteen (at least three times per month) or received food packages from their relatives (once per month).	Good (7/8)
**Ekmekcioglu 2015** ^71^ Austria 2001–2011	n = 43,992 M = 100% F = 0	WHO reference. Weight/height measured	40.1%	30% of the incarcerated individuals had overweight, 10% had obesity, and 2.8% had underweight. Incarcerated individuals arrested for the category fraud or sexual abuse of minors indicated higher mean BMI scores compared to the other categories. No data on dietary or PA was presented in the study.	Good (7/8)
**Leigey 2015** ^72^ United States 2013	n = 458 M = 0 F = 100%	CDC reference. Self‐reported weight/height measures	70.0%	34% of the incarcerated individuals had overweight, 36% had obesity, and no incarcerated individuals had underweight. These percentages were similar to the general population. Race and age were significantly (p < 0.05) associated with the BMI categories of healthy weight and obesity. No dietary or PA outcomes were reported in the study.	Good (7/8)
**Martínez‐Vicente 2014** ^73^ Spain 2013	n = 122 F = 81% M = 19%	Pi‐Sunyer, 2000 reference. Weight/height measured	56.6%	41.8% had overweight, 14.8% had obesity, and no incarcerated individuals had underweight. Prevalences were slightly below that of the Spanish population. No dietary or PA outcomes were reported in the study.	Poor (4/8)
**Nara 1998** ^75^ Japan 1995–1996	n = 400 M = 0 F = 100%	WHO reference. Weight/height measured	NR (only mean BMI presented)	The mean BMI of incarcerated individuals was 22.1 (±0.3). Dietary intake was restricted to 1800 kcal/day. Some incarcerated individuals were reported as active since some work at the factory for 7 hrs a day. Some others reported moderate exercise training for 30 min/day, walking around the prison grounds.	Moderate (6/8)
**Nwosu 2014** ^78^ United States 2010–2012	n = 526 M = 95% F = 5%	WHO reference. Weight/height measured	78.7%	42.8% of male incarcerated individuals and 33.3% of females had overweight, and 36.5% of males and 33.3% of females had obesity. No incarcerated individuals had underweight. Menus met or exceeded the recommended daily allowance for vitamin D of 600 IU. The vitamin D status of prison‐incarcerated individuals was determined by skin pigmentation, seasons, and the security level of incarceration. No data on PA was presented.	Good (7/8)
**Ross 2019** ^82^ Australia 2015	n = 1098 M = 68% F = 32%	Reference not reported. Self‐reported weight/height measures	17.3%*	17.3% of incarcerated individuals had obesity. 23.5% were defined as having low physical health status and a higher proportion of obesity (27.7% vs 13.9%). Those of low physical status also reported a higher risk of obesity (OR 2.38; 95%CI 1.54–3.66). Dietary intake was reported for fruit and vegetables. Those who had >1 serving of fruit per day (low vs high physical health status) 34.4% vs 46.6% (OR 0.60, 95%CI 0.45–0.80), and those that had >1 serving of vegetables per day (low vs high physical health status) 33.8% vs 34.1%. Less active now compared to before prison (low vs high physical health status) 56.8% vs 40.2% (OR 1.95, 95%CI 1.48–2.58).	Good (6/8)
**Sioen 2009** ^84^ Belgium 2007	n = 59 M = 100% F0	WHO reference. Weight/height measured	NR (only mean BMI presented)	Mean BMI was reported as 28.3 (±4.9). The results were reported as having no impact on the subject's waist circumference, weight, BMI, or systolic blood pressure. No dietary or PA outcomes were reported in the study**.**	Good (7/8)
**Togas 2014** ^85^ Greece 2013	n = 100 M = 100% F = 0	WHO reference. Weight/height measured	47.0%	34% of incarcerated individuals had overweight, 13% had obesity and no incarcerated individuals had underweight. No dietary or PA outcomes were reported in the study**.**	Good (8/8)
**Vera‐Remartínez 2014** ^86^ Spain 2013	n = 1077 M = 95% F = 5%	Reference not reported. Weight/height measured	51.9%	40.3% of male incarcerated individuals and 25.5% of females had overweight. 11.7% of male incarcerated individuals and 23.6% of females had obesity. No incarcerated individuals had underweight. Obesity was significantly associated with dyslipidemia (OR 1.54; 95%CI 1.00–2.36), hypertension (OR 2.04; 95%CI 1.32–3.15), diabetes (OR 4.18; 95%CI 2.35–7.44) and chronic disease (OR 3.85; 95%CI 2.50–5.930). All participants (38.5%) had a sedentary lifestyle, but when reported by sex, 37.9% of males and 50.9% of females had a sedentary lifestyle. No data on dietary intake were presented in the study.	Good (8/8)
**Voller 2016** ^87^ Italy 2014	n = 15,751 M = 94% F = 6%	WHO reference. Weight/height measured	48.3%	35.2% of incarcerated individuals had overweight, 13.1% had obesity and 1.9% had underweight. The prevalence of obesity was higher than in than the general population. No dietary or PA outcomes were reported in the study**.**	Good (8/8)
**Wright, 2019** ^89^ United Kingdom 2014–2015	n = 199 M = 100% F = 0	WHO reference. Weight/height measured	55.3%	46% of incarcerated individuals had overweight, 10% had obesity and no incarcerated individuals had underweight. 80% of incarcerated individuals ate fruit, 45% ate salad and/or vegetables and 41% ate salt daily.19% of incarcerated individuals did not perform any PA, while 22% did <150 minutes a week, and 59% did ≥150 minutes.	Good (7/8)
**Wolff 2012** ^90^ United States 2009	n = 4204 M = 95% F = 5%	WHO reference. Weight/height measured	78.3%	48.6% of male incarcerated individuals and 32.5% of female incarcerated individuals had overweight. 29.8% of male incarcerated individuals and 42.7% of females had obesity. No incarcerated individuals had underweight. No dietary or PA outcomes were reported in the study**.**	Good (7/8)
**Young 2005** ^91^ Australia 2002	n = 212 M = 0 F = 100%	WHO reference. Weight/height measured	44%	24% of incarcerated individuals had overweight, 20% had obesity and no incarcerated individuals had underweight. Prevalences for overweight and obesity were higher than that of the national population (24% and 15.8%, respectively). Only 10.2% of incarcerated individuals reported consuming the recommended daily intake of five or more servings of vegetables. In comparison, 40.7% reported consuming the recommended daily intake of two or more servings of fruit. Prevalence of sedentary life was double in incarcerated individuals compared with that in the community (p < 0.001).	Good (7/8)
**Maddan 2008** ^92^ United States 1970–2000	n = 5000 M = 100% F = 0	Reference not reported. Weight/height measured	27.8%	27.8% of the incarcerated individuals had a BMI > 26. The prisoner's somatotype was associated with criminal patterns, but this was a non‐significant predictor of criminality. No dietary or PA outcomes were reported in the study.	Good (7/8)
**Martínez‐Delgado 2016** ^93^ Spain 2014	n = 33 M = 100% F = 0	WHO reference. Weight/height measured	45.5%	33.3% of the incarcerated individuals had overweight, 12.1% had obesity and no incarcerated individuals had underweight. No dietary or PA outcomes were reported in the study.	Good (7/8)
**Saleh 2019** ^95^ United States 2009–2011	n = 362 M = 100% F = 0	WHO reference. Weight/height measured	30.6%*	30.6% of incarcerated individuals had obesity. 57% had two or more cardiovascular risk factors. Incarcerated individuals who self‐rated their health to be fair or poor had higher BMI, higher waist circumference, elevated triglyceride level, and low HDL. No dietary or PA outcomes were reported in the study.	Good (7/8)
**Packham 2020** ^96^ United Kingdom 2017–2019	n = 2107 M = 100% F = 0	WHO reference. Weight/height measured	NR (only mean BMI presented)	21.8% of incarcerated individuals had existing comorbidities and their mean BMI was 30.5 (6.7), while those without any comorbidity had a mean BMI of 26.9 (5.2). Cardiovascular risk was like that of the general population though they were ten years younger. No dietary or PA outcomes were reported in the study.	Good (7/8)
**Upper‐Middle‐Income Countries**					
**Audi 2018** ^18^ Brazil 2012–2013	n = 940 M = 0 F = 100%	WHO reference. Weight/height measured	47.0%	28.3% of the incarcerated individuals had overweight, 18.7% had obesity and no incarcerated individuals had underweight. A high prevalence of daily consumption of ultra‐processed foods was recorded among incarcerated individuals, and only 30% practised ≥30 minutes/day of PA. Also, consuming≥3 times/week vegetables was achieved by 50.5%; green leafy vegetables by 63.4%; fruits at 44.1%. A high frequency of daily consumption of natural or minimally processed foods such as rice, beans, and cassava flour were also reported.	Good (7/8)
**Rueda 2013** ^34^ Colombia 2010–2012	n = 1.233 M = 91% F = 9%	WHO reference. Weight/height measured	22.7%	19.1% of the incarcerated individuals had overweight, 3.6% had obesity and 5.1% had underweight. Being underweight was a risk factor associated with tuberculosis. All prisons were reported as overcrowded. No dietary or PA outcomes were reported in the study**.**	Good (7/8)
**Argüello‐González 2020** ^39^ México 2016–2017	n = 426 M = 100% F = 0	WHO reference. Weight/height measured	76.8%	51.4% of the incarcerated individuals had overweight, 25.3% had obesity and no incarcerated individuals had underweight. No dietary or PA outcomes were reported in the study.	Good (7/8)
**Bautista‐Arredondo 2015** ^48^ Mexico 2010	n = 17,084 M = 90% F = 10%	WHO reference. Weight/height measured	45.1%	32.4% of the male incarcerated individuals and 39.7% of the female had overweight. 9.5% of the male incarcerated individuals and 33.8% of the female had obesity. None of the incarcerated individuals had underweight. The prevalence of obesity was higher for females and lower for male participants compared to national statistics. 40.2% of males and 31.0% of females reported an increase in PA since incarceration; 21.3% and 20.2% reported similar levels of PA, and 38.5% and 48.9% reported less PA, respectively. No data on dietary outcomes were presented in this study.	Good (7/8)
**Lalem et al., 2015** ^69^ Libya NR	n = 87 M = 100% F = 0	WHO reference. Weight/height measured	26.4%	18.4% had overweight, 8% had obesity and 8% had underweight. No dietary or PA outcomes were reported in the study.	Good (7/8)
**Nessier 2012** ^76^ Argentina 2008–2009	n = 30 M = 0 F = 100%	WHO reference. Weight/height measured	63.3%	23.3% of the incarcerated individuals had overweight, 40.0% had obesity and 3.3% had underweight. The main nutritional problem reported was excess weight. Their dietary intake met the energy recommendations. 40% of the incarcerated individuals were sedentary.	Good (7/8)
**Lower‐Middle Income Countries**					
**Noeske 2011** ^6^ Cameroon 2009	n = 3219 M = 97% F = 3%	WHO reference. Weight/height measured	96.7%^§^	3.3% of the incarcerated individuals had underweight (BMI ≤ 18.5), and the rest (96.7%) had either normal weight or had overweight or obesity. No dietary or PA outcomes were reported in the study.	Moderate (6/8)
**Sharma 2020** ^33^ India NR	n = 181 M = 100% F = 0	WHO reference. Weight/height measured	16.6%	16.6% of the incarcerated individuals had overweight or had obesity, and 5.5% had underweight. No dietary or PA outcomes were reported in the study**.**	Good (7/8)
**Mukhtar 2013** ^37^ Pakistan NR	n = 269 M = 0 F = 100%	WHO reference. Weight/height measured	53.5%	26.4% of the incarcerated individuals had overweight, 27.1% had obesity and no incarcerated individuals had underweight. 75.4% of the incarcerated individuals had no access to fruits regularly; about 17.4% had fruits 1–3 servings a week, while 7.2% had fruit servings five or more days a week. Most of the incarcerated individuals who had access to fruits were foreigners. Overall, poor dietary habits were reported. No data of PA outcomes were presented	Good (7/8)
**Gould 2013** ^42^ Papua New Guinea 2010	n = 148 M = 100% F = 0	WHO reference. Weight/height measured	14.2%	14.2% of the incarcerated individuals had overweight or had obesity, and 4.7% had underweight. Most incarcerated individuals reported consuming fruit and vegetables rarely or never (66%), and 91% reported consuming these foods less than once per week. Incarcerated individuals' diets lacked several micronutrients. The median energy intake of the incarcerated individuals was 1775.8 kcals. 64% of the incarcerated individuals had low plasma vitamin C levels, 35% had low red blood cell folate concentrations, 65.7% had low serum zinc levels, 46% had low retinol levels, 7.6% had low ferritin levels and 79% had elevated levels of plasma homocysteine, with 35% having severe elevated levels	Good (7/8)
**Jimoh 2015** ^43^ Nigeria NR	n = 373 M = 99% F = 1%	WHO reference. Weight/height measured	17.7%	13.4% of the incarcerated individuals had overweight, 4.3% had obesity and 6.5% had underweight. No dietary or PA outcomes were reported in the study.	Good (7/8)
**Banu 2010** ^47^ Bangladesh 2005–2007	n = 11,000 M = 93% F = 7%	Reference not reported. Weight/height measured	4.6%	4.6% of the incarcerated individuals had overweight or had obesity, while 34% had underweight. No dietary or PA outcomes were reported in the study.	Good (7/8)
**Khodabakhshifard 2014** ^60^, ^67^ Iran 2008	n = 435 M = 89% F = 11%	WHO reference. Weight/height measured	5.8%	3.3% of male incarcerated individuals and 16.6% of females had overweight. 0.5% of male incarcerated individuals and 4.1% of females had obesity. 15.2% of male incarcerated individuals and 6.3% of females had underweight. Underweight was 2.5 times more prevalent in male incarcerated individuals than in the public population. Food intake was reported according to the food menu in prisons: grain (13.4 portions) 121%, protein food meat/beans (1 portion) 41%, vegetables (3.5 portions)116%, dairy (1.9 portion) 95% Fruit (0 portion) 0%. Fruit consumption was inadequate. 60% of the participants used the prison food menu and the others were able to buy food at the prison stores. 9.7% had low selenium levels. No data on PA were presented in the study.	Good (7/8)
**Himwaaba 2021** ^62^ Zambia 2020–2021	n = 311 M = 73% F = 27%	WHO reference. Weight/height measured	30.5%	22.8% of the incarcerated individuals had overweight, 7.7% had obesity and 7.1% had underweight. 54% of the incarcerated individuals were reported as having provisions for exercises such as sports and walking around. No dietary outcomes presented	Good (7/8)
**LaMonaca 2018** ^70^ Haiti 2011	n = 290 M = 100% F = 0	WHO reference. Weight/height measured	83%^§^	65% of the incarcerated individuals had high food insecurity. 17% had underweight. The length of incarceration was negatively associated with physical function (p = 0.004) and self‐rated health but positively associated with BMI (p < 0.05). Incarcerated individuals with more visitors were significantly (p < 0.05) less likely to be underweight. Food was supplied twice a day in a communal bucket. Some incarcerated individuals receive meals from family members and friends. No data on PA outcomes were presented in the study.	Moderate (6/8)
**Murad 2014** ^74^ Pakistan 2012–2013	n = 433 M = 100% F = 0	WHO reference. Weight/height measured	6.3%	5.1% of the incarcerated individuals had overweight, 1.2% had obesity and 8.3% had underweight. 37% of the incarcerated individuals had lost weight during incarceration. 9.0% of the incarcerated individuals had routine exercise and reported regularly going to the gym within the prison. 13.2% exercised but not regularly. Most of the incarcerated individuals (>68%) consumed food provided by prison authorities, and 24% used homemade and prison food. The diet of the incarcerated individuals was reported to be inadequate and nutritionally imbalanced.	Good (7/8)
**Noeske 2006** ^77^ Cameroon 2003–2004	n = 2474 M = 97% F = 3%	WHO reference. Weight/height measured	77.9%^§^	22.1% of the incarcerated individuals had underweight (BMI ≤ 18.5), and 77.9% had either normal weight, overweight, or obesity. No dietary or PA outcomes were reported in the study**.**	Moderate (6/8)
**Oyedokun 2018** ^79^ Nigeria 2017	n = 200 M = 95% F = 5%	WHO reference. Weight/height measured	11.0%	10.5% of the incarcerated individuals had overweight, 0.5% had obesity and 21.5% had underweight. The average energy intake (1794 kcal) was below recommendation. Over 70% of the incarcerated individuals consumed more cereal groups but little fruits and vegetables, meat, and poultry groups. The majority met over 70% of their RDA for energy and nutrients. The respondents consumed more of root and tubers, legumes, and cereals. About 37.5% of the incarcerated individuals perceived their health as good. There was a significant relationship between BMI and health perception (p = 0.001). No data on PA outcomes were presented in this study.	Good (7/8)
**Rahfiludin 2019** ^80^ Indonesia NR	n = 50 M = 0 F = 100%	Reference not reported. Weight/height measured	76.0%	22% of the incarcerated individuals had overweight, 54% had obesity and 2% had underweight. No dietary or PA outcomes were reported in the study**.**	Good (7/8)
**Simeni 2020** ^83^ Cameroon 2017	n = 437 M = 79% F = 21%	WHO reference. Weight/height measured	11.7%*	11.7% of the incarcerated individuals had obesity. 91.1% of the incarcerated individuals had a sedentary lifestyle. No dietary or PA outcomes were reported in the study**.**	Good (7/8)
**Winetsky 2014** ^88^ Tajikistan 2010	n = 1317 M = 100% F = 0	WHO reference. Weight/height measured	97%^§^	3% of the incarcerated individuals had underweight (BMI ≤ 18.5), and 97% had either a normal weight, overweight, or had obesity. Visits and deliveries from friends and family were associated with increased BMI (OR 1.15, p < 0.001, and OR 0.12, p < 0.001, respectively). No dietary or PA outcomes were reported in the study**.**	Good (7/8)
**Ullah 2010** ^94^ Pakistan NR	n = 166 M = 88% F = 12%	WHO reference. Weight/height measured	56.6%	34.9% had overweight, 21.7% had obesity and no incarcerated individuals had underweight. 12% of the incarcerated individuals had excess meat consumption (more than 500 g), while 50% had vegetables and 59% did not have fruits. 71.7% did not have regular exercise.	Good (7/8)
**Low‐Income Countries**					
**Kayomo 2018** ^35^ Democratic Republic of the Congo 2015	n = 610 M = NR F=NR	WHO reference. Weight/height measured	57.4%^§^	42.6% of the incarcerated individuals had underweight (BMI ≤ 18.5), and 57.4% had either a normal weight, overweight, or had obesity. Overcrowded living conditions and poor nutrition were the driving factors behind the high tuberculosis incidence. World Food Program assisted with supplementary feeding for the undernourished. No PA outcomes were presented in this study.	Moderate (5/8)
**Wondimu 2021** ^41^ Ethiopia 2020	n = 334 M = 95% F = 5%	WHO reference. Weight/height measured	81.4%^§^	18.6% of the incarcerated individuals had underweight (BMI ≤ 18.5), and 81.4% had either a normal weight, overweight, or had obesity. The prevalence of undernutrition in the prison was comparable to that in the general population. Only 22.5% consumed a varied diet (not necessarily nutritionally adequate), and 76.3% were physically inactive.	Moderate (6/8)
**Abera 2017** ^46^ Ethiopia 2017	n = 809 M = 97% F = 3%	WHO reference. Weight/height measured	75%§	25% of the incarcerated individuals had underweight (BMI ≤ 18.5), and 75% had either a normal weight, overweight, or had obesity. No dietary or PA outcomes were reported in the study.	Moderate (5/8)
**Diendéré 2021** ^56^ Burkina Faso 2017	n = 1004 M = 96% F = 4%	WHO reference. Weight/height measured	17.0%	17% of the incarcerated individuals had overweight or had obesity, and 8% had underweight. The food ration was composed of cereals (rice, cornflour, millet flour), legumes, and vegetables (beans, meat and fish, baobab leaves, dry okra, onion, amaranth, and garlic). No fruit was provided as part of the menu. Two meals were served per day (morning and noon). The nutritional value of the food ration was not reported. 30.1% had clinical beriberi. No data on PA outcomes were presented.	Good (7/8)
**Kalonji 2021** ^65^ Democratic Republic of the Congo 2019–2020	n = 300 M = 89% F = 11%	WHO reference. Weight/height measured	27.3%	27.3% of incarcerated individuals had overweight or had obesity, and 51.3% had underweight. The risk of developing severe malnutrition was higher for incarcerated individuals whose meals were only provided by the prison (OR 4.7; 95%CI 1.6–13.8). TB, HIV, and/or intestinal infections were also significantly associated with the risk of malnutrition (adjusted OR = 2.6 (1.4–4.7) Incarcerated individuals who had spent over 6 months in prison were reported to have up to 5 times higher risk of malnutrition than other incarcerated individuals. Although the food was provided once a day, it was reported to be insufficient in quantities and nutritional quality. No data on PA outcomes were presented in this study.	Moderate (6/8)
**Ravaoarisoa 2019** ^81^ Madagascar 2013	n = 125 M = 0 F = 100%	WHO reference. Weight/height measured	61.6%^§^	38.4% of the incarcerated individuals had underweight (BMI ≤ 18.5), and 61.6% had either a normal weight, overweight, or had obesity. Factors related to undernutrition were having two meals out of three meals a day (p = 0.003), insufficient energy intake (<1400kcals/day) (p < 0.001), incarceration duration of more than ten months (p < 0.001), absence of family visits (p = 0.013) and lack of financial assistance from family (p = 0.013). No data on PA were presented in this study.	Moderate (6/8)

### 3.1 Primary outcome

All 71 included studies reported on body weight‐related measurements; the results are summarized in Table 2. Overall, 68 studies had the weight and height of the participants objectively measured, while three studies used self‐reported measurements.^51^, ^54^, ^82^ Also, 58 studies used the WHO reference to categorize BMI, eight used the Centers for Disease Control and Prevention (CDC) reference,^13^, ^36^, ^38^, ^44^, ^49^, ^52^, ^53^, ^57^, ^72^ and five used other references (Table 2). Overall, the proportion of overweight and obesity within prisons and in comparison, with the national data, varied according to the sex of the participants. For example, incarcerated females had a higher reported proportion of overweight and obesity than males, or compared with national data. The proportion of overweight and obesity within carceral facilities, and in comparison, with the national data, also varied according to country income, with high‐income countries reporting a higher proportion of overweight and obesity (Table 2). Undernutrition was recorded in most studies; carceral facilities in low‐income countries recorded higher proportions of incarcerated individuals having underweight, with four^35^, ^46^, ^65^, ^81^ out of the six studies recording more than 25% of the incarcerated population classified as underweight (Table 2). Some studies reported health or social outcomes related to the BMI classification of incarcerated individuals (e.g., the association of obesity with painful previous experiences^52^). Furthermore, Beck et al (2013)^49^ reported that the population residing in prisons with overweight and obesity had significantly lower rates of incarcerated individuals‐on‐ incarcerated individuals sexual victimization and staff misconduct than those who were at or below normal weight. However, in one study assessing a population residing in jails, rates of incarcerated individuals‐on‐incarcerated individuals sexual victimization was almost double in those with underweight and in those with morbid obesity compared to other categories.^49^

**FIGURE 3:**
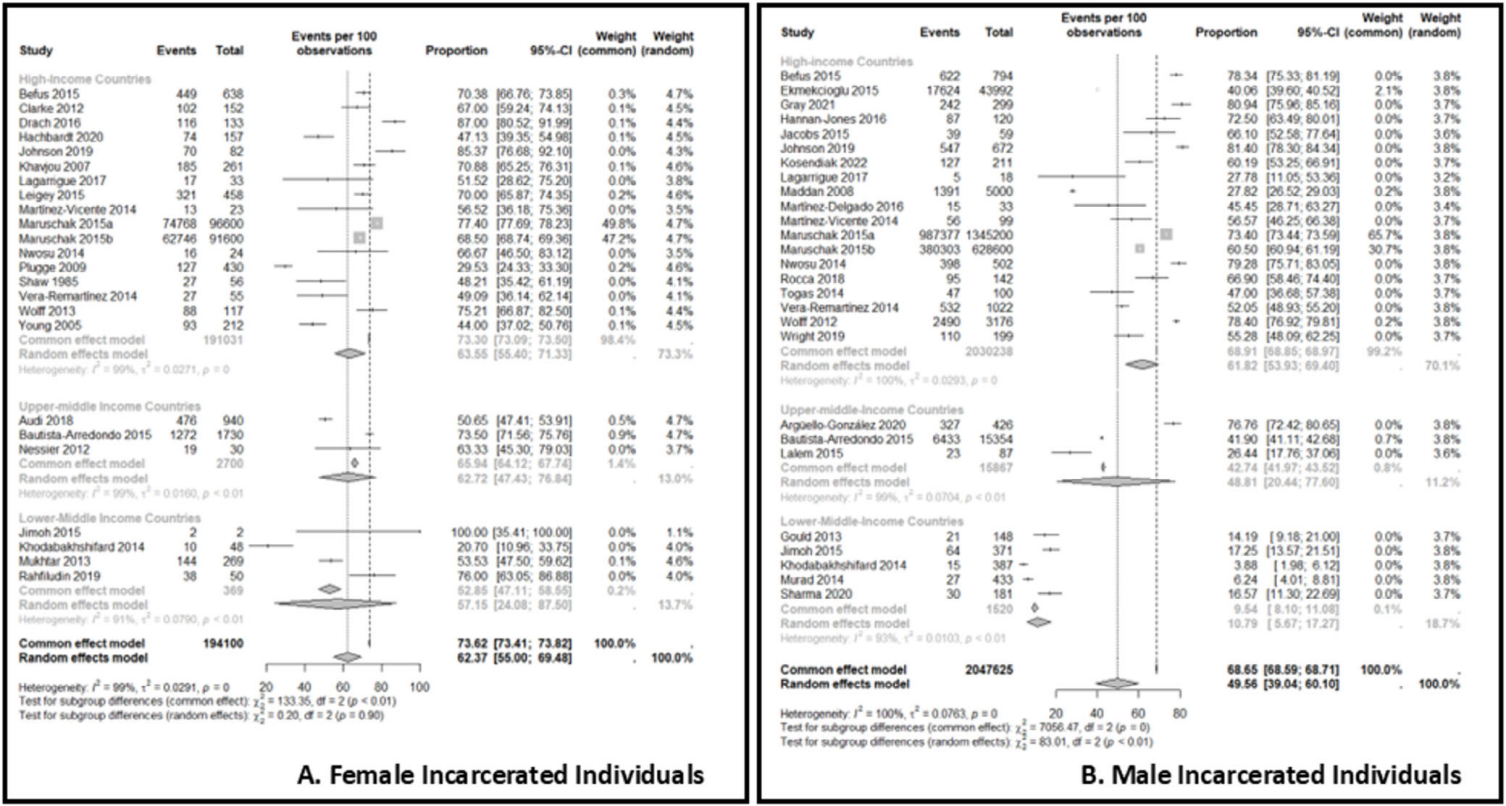
Prevalence of overweight and obesity in female (A) and male (B) incarcerated individuals. Events refer to the estimated prevalence of overweight and obesity in the included studies. The two vertical lines showcase the prevalence estimations according to the difference models (common vs random). Country income is categorized according to World Bank 2023 fiscal year income.

Thirty‐eight out of 71 studies reported sufficient data to estimate the proportion of incarcerated individuals with overweight and obesity within prisons and in comparison, with national data, and such data were pooled into a proportion meta‐analysis. For female incarcerated individuals, data from 23 studies,^11^, ^12^, ^18^, ^25^, ^36^, ^37^, ^40^, ^43^, ^44^, ^48^, ^50^, ^57^, ^60^, ^61^, ^64^, ^66^, ^67^, ^72^, ^76^, ^78^, ^80^, ^86^, ^90^, ^91^ comprising 194,100 incarcerated individuals, were pooled, and the overall proportion of female incarcerated individuals with overweight and obesity was 73.6% (95% CI 73.4, 73.8). However, studies in high‐income countries reported higher proportions of overweight and obesity (73.3%, 95% CI 73.1, 73.5) compared to upper‐middle‐income countries (66.1%, 95% CI 64.1, 67.7) and lower‐middle‐income countries (52.8%, 95% CI 47.1, 58.1) (Figure 3a).

For male incarcerated individuals, data from 26 studies,^12^, ^16^, ^17^, ^33^, ^36^, ^38^, ^39^, ^42^, ^43^, ^48^, ^50^, ^63^, ^64^, ^67^, ^68^, ^69^, ^71^, ^72^, ^73^, ^74^, ^78^, ^85^, ^86^, ^89^, ^90^, ^93^ comprising 2,047,625 incarcerated individuals, were pooled, and the overall proportion of male incarcerated individuals with overweight and obesity within prisons was 68.7% (95% CI 68.6, 68.7). Again, studies in high‐income countries reported higher proportions of overweight and obesity (68.9%, 95% CI 68.9, 69.0) compared to upper‐middle‐income countries (42.7%, 95% CI 42.0, 43.5) and lower‐middle‐income (9.5%, 95% CI 8.1, 11.1) (Figure 3b).

**FIGURE 4:**
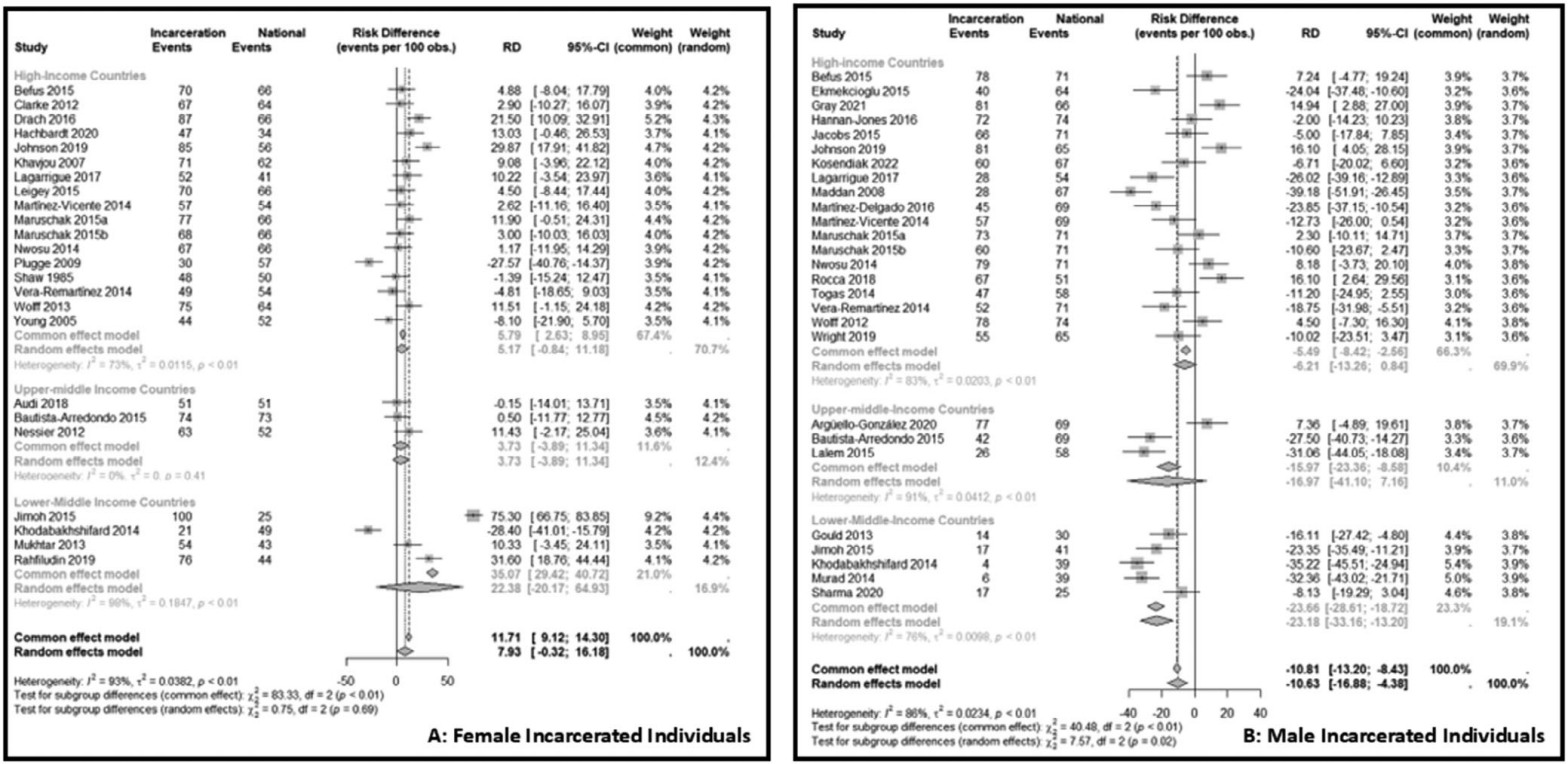
Prevalence of overweight and obesity in female (A) and male (B) incarcerated individuals. Prison refers to the estimated prevalence of overweight and obesity in incarcerated individuals, as reported in the included studies. National refers to the national overweight and obesity prevalence data, which was retrieved from the world obesity observatory for each country (adjusted for gender and year of data collection) of the studies included in the meta‐analyses. Country income is categorized according to World Bank 2023 fiscal year income. The continuous line represents no difference in the observed risk of events between prevalence of overweight and obesity among incarcerated individuals compared with the national prevalence in each country. The dashed lines represent the relative increase in the prevalence of overweight and obesity among incarcerated individuals compared with the national prevalence in each country (estimated by common and random models).

In addition, we compared the estimated proportions of overweight and obesity in the incarcerated population with those reported for the general population in each of the countries. The overall proportion of overweight and obesity in female incarcerated individuals was higher than that in the general population (RD 11.7%, 95% CI 9.1, 14.3). However, this difference was more pronounced for lower‐middle‐income countries (RD 35.1%, 95% CI 29.4, 40.7) than for high‐income countries (RD 5.8%, 95% CI 2.6, 9.0) or upper‐middle‐income countries (RD 3.7%, 95% CI ‐3.9, 11.3) (Figure 4a). In contrast, the overall proportion of overweight and obesity in male incarcerated individuals was lower than that in the general population in all categories (RD ‐10.8%, 95%CI ‐13.2, −8.4) across different country income levels. The difference between prison and general populations was more pronounced for lower‐middle‐income countries (RD ‐23.7%, 95% ‐28.6, −18.7) than for high‐income countries (RD ‐5.9%, 95% CI ‐8.4, −2.6) or upper‐middle‐income countries (RD ‐16.0%, 95% CI ‐23.4, −8.6) (Figure 4b).

### 3.2 Secondary outcomes

Dietary intakes were reported in 26 studies,^17^, ^18^, ^19^, ^25^, ^37^, ^40^, ^41^, ^42^, ^55^, ^56^, ^57^, ^58^, ^64^, ^65^, ^67^, ^68^, ^70^, ^74^, ^75^, ^76^, ^79^, ^81^, ^82^, ^89^, ^91^, ^94^ physical activity outcomes were reported in 20 studies,^12^, ^16^, ^17^, ^18^, ^19^, ^25^, ^41^, ^48^, ^55^, ^57^, ^61^, ^62^, ^64^, ^68^, ^74^, ^75^, ^76^, ^86^, ^89^, ^91^, ^94^ and micronutrient deficiencies reported in five studies.^42^, ^56^, ^60^, ^63^, ^78^ Overall, dietary and physical activity outcomes were measured using different assessment tools. For example, ten studies^18^, ^19^, ^25^, ^37^, ^64^, ^68^, ^79^, ^81^, ^89^, ^91^ used Food Frequency Questionnaires (FFQ), two used food diaries,^55^, ^75^ three used a 24‐hour recall,^41^, ^42^, ^76^ three used interviews,^65^, ^74^, ^82^ two used prison menus^40^, ^67^ and five used other means.^17^, ^56^, ^57^, ^58^, ^70^ Four studies that reported energy intake in high‐income and upper‐middle‐income countries found that the study population achieved dietary guidelines. In contrast, two studies from the United States^40^ and the United Kingdom^55^ found that the incarcerated individuals exceeded the dietary recommendations for energy intake. In lower‐middle‐income and low‐income countries, five of the six studies reported that the energy intake of incarcerated individuals was below the recommended dietary guidelines, whereas one study did not quantify an average dietary intake due to the serving system; food was served collectively in buckets without individual portions. Nine studies from high‐income and upper‐middle‐income countries reported fruit and vegetable intake among incarcerated individuals; average portions were within dietary recommendations. Six studies from lower‐middle‐income and low‐income countries reported fruit and vegetable intake. Of these, two studies reported that incarcerated individuals were not given access to fruits, while the other four recorded inadequate intake of fruit and vegetables (Table 3). Generally, incarcerated individuals in high‐income and upper‐middle‐income countries had access to food from prison canteens, cafeterias, or food stores on top of their prison rations. In lower‐middle‐income and low‐income countries, incarcerated individuals depended on prison rations or visitors for food.

**TABLE 3:** Summary of dietary and physical activity results.

**STUDY ID** **Country**	**Sample** Sample size (n) Male (%) Female (%)	Dietary intake				Physical activity	
		Assessment tool	Recommended total energy intake^a^	% of incarcerated individuals consuming fruit % of incarcerated individuals consuming vegetables	Number of eating occasions per day Source of food	Assessment tool	% of incarcerated individuals achieving WHO recommendations for physical activity ^b^
**High‐Income Countries**							
**Johnson 2018** ^11^, ^64^ Canada 2016–2017	n = 754 M = 89% F = 11%	FFQ	NR	75% ate ≥1 fruit a day. 70% ate ≥1 vegetable serving	NR Prison rations and canteen	IPAQ	58%
**Lagarrigue 2017** ^12^ France	n = 51 M = 35% F = 65%	NA	NA	NA	NA	Self‐reported questionnaire	Male 88.2% Female 62.1%
**Gray 2021** ^16^ United Kingdom	n = 299 M = 100% F = 0	NA	NA	NA	NA	Self‐reported questionnaire	48.7%
**Hannan‐Jones 2016** ^17^ Australia	n = 120 M = 100% F = 0	Diet history, previous menu reviews, and food purchase records	**↑**	NR	NR A no‐choice menu had additional self‐funded snacks and beverages	Self‐reported questionnaire	84%
**Nucci 2020** ^19^ Italy	n = 22 M = 50% F = 50%	FFQ	NR	37.2% > seven times a week.	NR Prison rations or buy from an approved list	Self‐reported questionnaire	45.3%
**Plugge 2009** ^25^ United Kingdom	n = 430 M = 0 F = 100%	FFQ	NR	12% met five a day.	NR NR	Self‐reported questionnaire	11%
**Shaw 1985** ^40^ United States	n = 56 M = 0 F = 100%	Prison food menu.	**↑**	100% 100%	NR Prison rations and canteens	NA	NA
**Choudhry 2019** ^55^ United Kingdom	n = 367 M = 100% F = 0	Food diaries	74% **↑** 26% **↔**	NR	NR Prison rations and canteen	Self‐reported questionnaire	84%
**Drach 2016** ^57^ United States	n = 133 M = 0 F = 100%	Structured interview	NR	NR	NR	Self‐reported questionnaire	85%
**Edwards 2001** ^58^, ^59^ United Kingdom	n = 506 M = 100% F = 0	Modified visual estimation technique	**↔**	fruit was given as an option for dessert. 100%	3 Prison rations and canteen	NA	NA
**Hachbardt 2020** ^61^ Italy	n = 157 M = 0 F = 100%	NA	NA	NA	NA	Self‐reported physical activity	8.5%
**Kosendiak 2022** ^68^ Poland	n = 211 F = 100% M = 0	FFQ	NR	NR	3–5 Prison rations, canteen, and visitors (families/friends)	IPAQ	55.9%
**Nara1998** ^75^ Japan	n = 400 M = 0 F = 100%	Food diary	**↔**	NR	NR Prison rations only	Self‐reported questionnaire	100%
**Ross 2019** ^82^ Australia	n = 1098 M = 68% F = 32%	Structured interview	NR	40.5% 34%	NR NR	NA	NA
**Vera‐Remartínez 2014** ^86^ Spain	n = 1077 M = 95% F = 5%	NA	NA	NA	NA	Self‐reported questionnaire	Male 62.1% Female 50.9%
**Wright 2019** ^89^ United Kingdom	n = 199 M = 100% F = 0	FFQ	NR	80% 45%	NR Prison rations and canteens	Self‐reported questionnaire	59%
**Young 2005** ^91^ **Australia**	n = 212 M = 0 F = 100%	FFQ	NR	40.7% met recommended daily intake fruit 10.2% met recommended daily intake of vegetables	NR NR	Self‐reported questionnaire	47.8%
**Upper‐Middle‐Income Countries**							
**Audi 2018** ^18^ Brazil	n = 940 M = 0 F = 100%	FFQ	NR	44.1%. 63.4% ate green‐leafy vegetables 50.5% ate other vegetables	>3 Prison rations, canteen, and visitors	Self‐reported questionnaire	30%
**Bautista‐Arredondo 2015** ^48^ Mexico	n = 17,084 M = 90% F = 10%	NA	NA	NA	NA	GPAQ‐2	Male 60.4% Female 52.3%
**Nessier 2012** ^76^ Argentina	n = 30 M = 0 F = 100%	24‐hour recall	**↔**	NR	NR NR	Self‐reported questionnaire	60%
**Lower‐Middle Income Countries**							
**Mukhtar 2013** ^37^ Pakistan	n = 269 M = 0 F = 100%	FFQ	NR	29.4% NR	NR Self and prison rations	NA	NA
**Gould 2013** ^42^ Papua New Guinea	n = 148 M = 100% F = 0	24‐hour recall	**↓**	66% < once a week 91% < once a week	>3 Prison rations + visitors		
**Khodabakhshifard 2014** ^60^, ^67^ Iran	n = 435 M = 89% F = 11%	Prison food menu.	**↓**male **↔** female	No fruit was provided to incarcerated individuals 100%	NR Prison food and foodstuff of the prison stores	NA	NA
**Himwaaba 2021** ^62^ Zambia	n = 311 M = 73% F = 27%	NA	NA	NA	NA	Self‐reported questionnaire	54%
**LaMonaca 2018** ^70^ Haiti	n = 290 M = 100% F = 0	Four‐item Likert scale	NR	NR	2 Prison rations and visitors (family and friends)	NA	NA
**Murad 2014** ^74^ Pakistan	n = 433 M = 100% F = 0	Structured interview	NR	NR	NR Homemade and prison food	Self‐reported questionnaire	22.2%
**Oyedokun 2018** ^79^ Nigeria	n = 200 M = 95% F = 5%	FFQ, 24‐hour recall	**↓**	63.7% met >1 a week. 90%	NR NR	NA	NA
**Ullah 2010** ^94^ Pakistan	n = 166 M = 88% F = 12%	Structured interview	NR	41% 50%	NR NR	Self‐reported questionnaire	28.3%
**Low‐Income Countries**							
**Wondimu 2021** ^41^ Ethiopia	n = 334 M = 95% F = 5%	24‐hour recall	NR	NR	1–4 Prison rations and visitors	GPAQ	23.7% for both (male and female)
**Diendéré 2021** ^56^ Burkina Faso	n = 1004 M = 96% F = 4%	Local food composition tables	Unable to measure	No fruit was provided to incarcerated individuals. Vegetables were provided	2 Prison rations	NA	NA
**Kalonji 2021** ^65^ Democratic Republic of the Congo	n = 300 M = 89% F = 11%	Interviews	**↓**	NR	1 Prison, family, or NGO	NA	NA
**Ravaoarisoa 2019** ^81^ Madagascar	n = 125 M = 0 F = 100%	FFQ	**↓**	NR	2 (83%), 3(17%) Prison rations and visitors	NA	NA

Seventeen studies^12^, ^16^, ^17^, ^18^, ^19^, ^25^, ^48^, ^55^, ^57^, ^61^, ^64^, ^68^, ^75^, ^76^, ^86^, ^89^, ^91^ from high‐income and upper‐middle‐income countries reported physical activity, with 11 showing more than half the population achieving the recommended levels. However, three out of four studies from lower‐middle‐income and low‐income countries recorded that less than 30% of the incarcerated individuals achieved the recommendations (Table 3).

Few studies reported on biomarkers of nutritional status. Two studies from high‐income countries reported that more than half of the incarcerated population was deficient in vitamin D.^63^, ^78^ One study reported that 64% of the incarcerated population had low plasma vitamin C levels, 46% had low retinol levels, 35% had low red blood cell folate concentrations, 65.7% had low serum zinc levels, 7.6% had low ferritin levels and 79% had elevated levels of plasma homocysteine (with 35% having severely elevated levels).^42^ Another study reported that 9.7% (7.8% of males and 25.5% of females) had low serum selenium levels^60^ while the third study reported that 30.1% of the incarcerated population had clinical beriberi,^56^ caused by vitamin B1 deficiency.

### 3.3 Quality of studies critical appraisal

Overall, 54 of the included studies were considered good quality, 14 of moderate quality, while only three were considered poor quality (Table 2, full appraisal shown in Table S2). All the studies identified confounders, while only seven reported on how they considered confounders in the analysis. Studies from low‐income countries had lower quality scores than those in higher‐income countries. In addition, all studies from low‐income countries were unclear on how they categorized weight‐related outcomes since they focussed on communicable diseases and reported on those who had underweight only.

## 4 DISCUSSION

This review systematically assessed body weight‐related outcomes in incarcerated populations in developing and developed countries, as well as possible determinants of these outcomes, including dietary intake and physical activity. Seventy‐one studies were included in this systematic review, with the majority performed in high‐income countries. The overall proportion of incarcerated individuals with overweight and obesity was highest in high‐income countries. Compared to national prevalences, incarcerated females had a higher prevalence, and incarcerated males had a lower prevalence of overweight and obesity, especially in lower‐middle‐income countries. Across studies, incarcerated individuals in high‐income and upper‐middle‐income countries were reported to meet or exceed their countries' recommended energy intake. In most cases, they had provision of fruit and vegetables. Also, a greater proportion of incarcerated individuals, male mostly, in high‐income and upper‐middle‐income countries met the WHO recommendations on physical activity levels.

Overweight and obesity are associated with an increased risk of metabolic diseases such as Type 2 Diabetes Mellitus, cardiovascular diseases like hypertension and stroke, diseases affecting bones and muscles like osteoarthritis, and some cancers that affect the digestive and reproductive system.^22^ Most reviews on overweight and obesity in incarcerated individuals have been conducted in high‐income and upper‐middle‐income countries.^8^, ^9^, ^21^, ^97^, ^98^ At the same time, there are currently no studies that have assessed prevalences of overweight and obesity by country income or by region. Our study revealed that female incarcerated individuals, especially those who are living in low‐middle‐income countries, had a higher proportion of overweight and obesity when compared to national data obtained for the general population, which coincides with a previous review.^9^ Despite low‐middle‐income countries often reporting higher proportions of undernutrition, our review suggests that there may be an emerging trend of higher prevalences of overweight and obesity among female incarcerated individuals in these countries. These findings relate to a recent report indicating that low‐middle‐income countries are increasingly exposed to the double burden of malnutrition, including under‐nutrition and overnutrition.^20^, ^99^ Undernutrition may lower immunity and increase the risk of communicable diseases like tuberculosis that are prevalent in carceral facilities.^100^ At the same time, overnutrition has been linked to increasing the risk of having overweight and obesity and diet‐related non‐communicable diseases like Type 2 Diabetes Mellitus, cardiovascular disease, and selected cancers,^22^ which add a higher economic burden to the carceral facilities budgets for health and medical care.^24^

The proportion of overweight and obesity in male incarcerated individuals varied according to income levels, with the high‐income countries showing the highest proportions. Compared with the general population, the incarcerated population had lower proportions of overweight and obesity in all income categories, albeit the overall prevalence of obesity in incarcerated individuals was still considered high, especially in high‐income countries. Our results concur with overweight and obesity proportions in two previous reviews.^9^, ^21^ In contrast, an Australian study found that the prevalence of cardiovascular risk factors in younger incarcerated individuals was higher than in the general population.^15^ A study from the United Kingdom found that younger male incarcerated individuals had higher risks of cardiometabolic disease.^16^ Globally, epidemiological studies and surveys have shown that metabolic disease prevalence has been escalating in incarcerated populations.^3^, ^36^, ^43^, ^54^, ^61^ Additionally, in high‐income and upper‐middle‐income countries, longer duration spent on incarceration has also been linked to increased BMI,^44^, ^55^ while higher BMI has been associated with increased susceptibility to infections caused by *Staphylococcus aureus*,^50^ creating concern about nutrition and health care within carceral facilities.

In this review, we considered dietary and physical activity outcomes as possible determinants of prevalence levels of overweight and obesity. Intake of a healthy diet has been associated with reducing all forms of malnutrition, including undernutrition, over‐nutrition, and micronutrient deficiencies.^31^ Nutritional requirements of an individual are influenced by many factors like sex, age, and level of activity, among others.^101^, ^102^ However, excess energy intake over a period of time may lead to overnutrition.^101^ Globally, observational studies and reports indicate that the majority of carceral facilities tend to serve the same amount of food to both male and female incarcerated individuals.^59^, ^67^, ^103^, ^104^, ^105^, ^106^ Therefore, female incarcerated individuals are likely to consume excess calories from the food served within the carceral facilities.^59^, ^67^, ^103^, ^105^ Indicatively, higher proportions of overnutrition have been reported in female than male incarcerated individuals.^12^, ^36^, ^48^, ^67^, ^107^ As shown by our results, the proportion of incarcerated individuals achieving WHO recommendations for physical activity is different among countries and among sex, with incarcerated males from high‐income countries being more active. However, it is important to note that across the studies included in this review, the number of studies considering incarcerated women as participants was lower compared to those including incarcerated men. More studies are needed that consider the impact of diets and physical activity on incarcerated women and their lifestyles.

Dietary intake in incarcerated populations varied across studies and according to the income level of the country. In most carceral facilities in high‐income and upper‐middle‐income countries, the diet of incarcerated individuals was based on nutritional needs, depending on the prisoner's age, physiological state, and religious and cultural preferences, with some carceral facilities having a budget allocation per incarcerated individual.^11^, ^19^, ^58^, ^68^ Most high‐income and upper‐middle‐income countries achieved the recommended energy intake, with a few exceeding their national guidelines for dietary intake. Studies from low‐middle‐income and low‐income countries showed that incarcerated individuals had lower‐quality diets, as their total energy intake was lower than those recommended by dietary guidelines, and/or they provided the same amount of food to male and female incarcerated individuals.^67^, ^79^ One study found that female incarcerated individuals achieved their energy requirements and had higher proportions of overweight and obesity than the male incarcerated individuals, whose energy intake was lower than the recommended and had a higher proportion of underweight.^67^ Similarly, a study in Ghana reported that, on average, female incarcerated individuals had excess energy intake compared to their recommended daily allowance and had more food than male incarcerated individuals.^103^ Undernourishment among incarcerated individuals in developing countries could be linked to the duration spent in the institution or could even be a pre‐existing condition. However, the latter is difficult to assess with the evidence retrieved, as most of the studies included in this review have a cross‐sectional design. Nevertheless, some studies have revealed that staying longer in carceral facilities increased the risk of undernutrition with some indicating drastic weight loss after a duration in incarceration.^67^, ^74^ In developed countries, weight gain and overnutrition among the incarcerated individuals was observed after a longer stay in the facilities.^107^

Generally, most diets in low‐middle‐income countries and low‐income countries do not consider individual nutritional needs. Some studies, especially those in high‐income countries, reported that incarcerated individuals had access to more food outlets (e.g., stores, canteens, or from visitors). Whilst prison rations in some countries provide adequate dietary intake, incarcerated individuals can purchase extra food items to increase their energy intake, especially if they are high in energy, fat, and sugar.^17^ Most prisons in low‐middle‐income and low‐income countries relied mainly on prison food rations, which were deemed inadequate, and to some extent, food from visitors was allowed. Only one study in the low‐middle‐income and low‐income category reported access to prison canteens, cafeterias, or food stores.^67^ We observed that prisons with canteens, cafeterias, or food stores also recorded higher proportions of overweight and obesity.^11^, ^18^, ^19^, ^55^, ^89^ Access to canteens, cafeterias or food stores could influence the frequency of eating occasions, which, if not monitored, could lead to excess energy intake that can lead to weight gain.^22^ Indeed, this was evident in studies that reported a significant correlation between weight gain and food items bought from the canteens, cafeterias, or food stores,^11^ and the fact that snacks bought by incarcerated individuals increased their energy intake.^17^

Fruits and vegetables provide essential nutrients like vitamin C, promoting health and enhancing the diet's nutritional quality.^108^ In this review, prison rations in high‐income and upper‐middle‐income countries had provision for fruit and vegetables, with higher proportions of the prison population consuming these, coinciding with previous reports which showed that the diet of incarcerated individuals in the United States,^105^ England,^59^ Canada,^11^ Poland,^68^, ^109^ and Italy^19^ provided a variety of fruit and vegetables. On the other hand, prisons in low‐middle‐income and low‐income countries had minimal provision of fruit and vegetables, with some countries not providing any fruit.^56^, ^67^ These results are consistent with other studies from low‐middle‐income countries that reported no fruit provision in the prison ration.^103^, ^110^, ^111^ Mukhtar (2021)^37^ reported that low fruit and vegetable intake was linked to a higher prevalence of overweight and obesity. Additionally, previous case studies in prisons from low‐middle‐income and low‐income countries have reported scurvy, a disease caused by insufficient vitamin C,^112^, ^113^ and one study reported on vitamin A deficiency among incarcerated individuals and cases of xerophthalmia, a disease caused by vitamin A deficiency.^114^ A study in Papua New Guinea revealed that a higher proportion of the prison population was at risk of scurvy and xerophthalmia as 64% of the incarcerated individuals had low plasma vitamin C levels and 46% had low serum retinol levels. In this study, more than half of the incarcerated individuals had low red blood cell folate concentrations and low serum zinc levels, and over three‐quarters of incarcerated individuals had elevated levels of plasma homocysteine.^42^ Another study in Burkina Faso reported that more than 30% of the incarcerated individuals had clinical beriberi, a disease caused by low vitamin B1.^56^ To improve the dietary intake of incarcerated individuals in these countries, the inclusion of different food groups like fruit and vegetables and whole grains should be considered as part of the prison rations.

Engaging in physical activity has been associated with reducing the rates of overweight and obesity and reducing the risks of non‐communicable diseases.^31^, ^64^ High‐income and upper‐middle‐income countries had more incarcerated individuals meeting the WHO recommendations for physical activity.^31^ Also, in high‐income and upper‐middle‐income countries, male incarcerated individuals were reported to be more active than female incarcerated individuals, possibly because physical activity among female incarcerated individuals is less supported. This may explain the higher prevalence of overweight and obesity in this group compared to male prisoners.^12^, ^48^, ^86^ In a previous review, supervised physical activities among incarcerated individuals were reported to have a beneficial effect on reducing cardiovascular risk and improving mental health.^115^ Additionally, increased physical activity has been linked to improving the physical and mental domains of incarcerated individuals' quality of life (QoL).^116^ In contrast, lower‐middle‐income and low‐income countries recorded lower proportions of incarcerated individuals meeting the WHO recommendations for physical activity. Reports indicated that congestion in prison and inadequate space for exercise did not enable the incarcerated individuals to engage in physical activity regularly.^41^

The strengths of this review include being the first to analyze prevalences of overweight and obesity in incarcerated individuals in relation to country income level. We followed a strict review process to ensure the data from different regions were retrieved. Such data were scrutinized carefully, and quality was appraised and included in the synthesis. In addition, a meta‐analysis was conducted to provide a more robust comparison of available data on overweight and obesity prevalences in incarcerated individuals globally. Limitations include that most studies were from high‐income and upper‐middle‐income countries, and such studies had better quality compared to lower‐income countries' studies, which may have affected the comparison between studies. Also, it would have been better if comparison with the general population would be according to social class grouping, especially in developed countries where people that are most deprived are faced with a higher number of cases of obesity and incarceration. Finally, it should be noted that high BMI, whilst widely used as an indicator of overweight and obesity, could indicate higher muscle mass in some incarcerated individuals.

In conclusion, the review shows that prevalence of overweight and obesity in populations differs between developed and developing countries. Incarcerated individuals from high‐income and upper‐middle‐income countries had higher proportions of overweight or obesity than those in low‐middle‐income countries, while female incarcerated individuals across all country income groups had higher proportions of overweight and obesity than those found in the general population. especially in lower‐middle‐income countries. Prevalences are likely to depend on factors such as dietary intake and physical activity. Our review highlights the need for more high‐quality studies on the prevalence of overweight and obesity, and on dietary intake, in low‐middle‐income and low‐income countries, and a stronger focus on managing overweight and obesity in female incarcerated individuals, especially in lower‐middle‐income countries.

## AUTHOR CONTRIBUTIONS

LM, AMJ, and BdR and designed and conceptualized the work. LM and MA‐M performed data collection, and were responsible for the analysis; all authors took part in the interpretation of results and the writing of the manuscript.

## CONFLICT OF INTEREST STATEMENT

The authors have no conflict of interest to report.

## Supporting information


**Table S1.** Search strategy.
**Table S2.** Risk of Bias Critical Appraisal.

## References

[1] Institute for Crime & Justice Policy Research . I. Prison populations continue to rise in many parts of the world, new report published by the Institute for Crime & Justice Policy Research shows. |Institute for Criminal Policy Research. Published December 1, 2021. Accessed July 6, 2022. https://icpr.org.uk/news-events/2021/prison-populations-continue-rise-many-parts-world-new-report-published-institute

[2] United Nations Office on Drugs and Crime . The United Nations Standard Minimum Rules for the Treatment of Prisoners. United Nations; 2015:38.

[3] World Health Organization . Addressing the Noncommunicable Disease (NCD) Burden in Prisons in the WHO European Region: Interventions and Policy Options. World Health Organization. Regional Office for Europe; 2022.

[4] Lines R . The right to health of prisoners in international human rights law. Int J Prison Health. 2008;4(1):3‐53. doi:10.1080/17449200701862145 18382849

[5] Puglisi LB , Wang EA . Health care for people who are incarcerated. Nat Rev Dis Primers. 2021;7(1):50. doi:10.1038/s41572-021-00288-9 34238928

[6] Noeske J , Ndi N , Mbondi S . Controlling tuberculosis in prisons against confinement conditions: a lost case? Experience from Cameroon. Int J Tuberc Lung Dis. 2011;15(2):223‐227. 21219685

[7] Min J , Zhao Y , Slivka L , Wang Y . Double burden of diseases worldwide: coexistence of undernutrition and overnutrition‐related non‐communicable chronic diseases: global double burden of diseases. Obes Rev. 2018;19(1):49‐61. doi:10.1111/obr.12605 28940822 PMC5962023

[8] Bondolfi C , Taffe P , Augsburger A , et al. Impact of incarceration on cardiovascular disease risk factors: a systematic review and meta‐regression on weight and BMI change. BMJ Open. 2020;10(10):e039278. doi:10.1136/bmjopen-2020-039278 PMC7569938 33067292

[9] Herbert K , Plugge E , Foster C , Doll H . Prevalence of risk factors for non‐communicable diseases in prison populations worldwide: a systematic review. The Lancet. 2012;379(9830):1975‐1982. doi:10.1016/S0140-6736(12)60319-5 22521520

[10] Al‐Rousan T , Rubenstein L , Sieleni B , Deol H , Wallace RB . Inside the nation's largest mental health institution: a prevalence study in a state prison system. BMC Public Health. 2017;17(1):342. doi:10.1186/s12889-017-4257-0 28427371 PMC5397789

[11] Johnson C , Chaput JP , Rioux F , Diasparra M , Richard C , Dubois L . An exploration of reported food intake among inmates who gained body weight during incarceration in Canadian federal penitentiaries. PLoS ONE. 2018;13(12):e0208768. doi:10.1371/journal.pone.0208768 30562361 PMC6298656

[12] Lagarrigue A , Ajana S , Capuron L , Féart C , Moisan MP . Obesity in French inmates: gender differences and relationship with mood, eating behavior and physical activity. PLoS ONE. 2017;12(1):e0170413. doi:10.1371/journal.pone.0170413 28103297 PMC5245834

[13] Gates ML , Bradford RK . The impact of incarceration on obesity: are prisoners with chronic diseases becoming overweight and obese during their confinement? J Obes 2015;2015:532468. 10.1155/2015/532468 25866674 PMC4381682

[14] Bai JR , Befus M , Mukherjee DV , Lowy FD , Larson EL . Prevalence and predictors of chronic health conditions of inmates newly admitted to maximum security prisons. J Correct Health Care. 2015;21(3):255‐264. doi:10.1177/1078345815587510 26084947 PMC4491502

[15] D'Souza RM , Butler T , Petrovsky N . Assessment of cardiovascular disease risk factors and diabetes mellitus in Australian prisons: is the prisoner population unhealthier than the rest of the Australian population? Aust N Z J Public Health. 2005;29(4):318‐323. doi:10.1111/j.1467-842x.2005.tb00200.x 16222927

[16] Gray BJ , Craddock C , Couzens Z , et al. Abundance of undiagnosed cardiometabolic risk within the population of a long‐stay prison in the UK. Eur J Public Health. 2021;31(3):461‐466. doi:10.1093/eurpub/ckaa187 33057683

[17] Hannan‐Jones M , Capra S . What do prisoners eat? Nutrient intakes and food practices in a high‐secure prison. Br J Nutr. 2016;115(8):1387‐1396. doi:10.1017/S000711451600026X 26900055

[18] Audi C , Santiago S , Andrade M , et al. Ultra‐processed foods consumption among inmates in a women's prison in São Paulo, Brazil. Rev Esp Sanid Penit. 2018;20(3):87‐94. Accessed March 14, 2022. Accessed https://www.ncbi.nlm.nih.gov/pmc/articles/PMC6463322/ 30908571 PMC6463322

[19] Nucci D , Licitra L , Sciara S , Moretti M , Gianfredi V . Prunus: design and validation of a questionnaire among prisoners – data of pilot study in the Penitentiary Institute of Perugia, Italy. Int J Prison Health. 2020;16(2):165‐183. doi:10.1108/IJPH-01-2019-0001 32378833

[20] Wells JC , Sawaya AL , Wibaek R , et al. The double burden of malnutrition: aetiological pathways and consequences for health. The Lancet. 2020;395(10217):75‐88. doi:10.1016/S0140-6736(19)32472-9 PMC7613491 31852605

[21] Choudhry K , Armstrong D , Dregan A . Systematic review into obesity and weight gain within male prisons. Obes Res Clin Pract. 2018;12(4):327‐335. doi:10.1016/j.orcp.2018.02.003 29478832

[22] World Health Organisation W . Obesity and overweight. Published June 1, 2021. Accessed July 6, 2022. https://www.who.int/news-room/fact-sheets/detail/obesity-and-overweight

[23] Alberti KGMM , Zimmet P , Shaw J . Metabolic syndrome—a new world‐wide definition. A consensus statement from the International Diabetes Federation. Diabet Med. 2006;23(5):469‐480. doi:10.1111/j.1464-5491.2006.01858.x 16681555

[24] Agozino B , Volpe SL . Health inequalities in correctional institutions: implications for health inequalities in the community. J Correct Health Care. 2009;15(4):251‐267. doi:10.1177/1078345809333407 19633333

[25] Plugge EH , Foster CE , Yudkin PL , Douglas N . Cardiovascular disease risk factors and women prisoners in the UK: the impact of imprisonment. Health Promot Int. 2009;24(4):334‐343. doi:10.1093/heapro/dap034 19854844

[26] World Bank . World Bank country and lending groups – World Bank data help desk. Published July 2021. Accessed July 11, 2022. https://datahelpdesk.worldbank.org/knowledgebase/articles/906519-world-bank-country-and-lending-groups

[27] Food and Agriculture Organization . Home|Food‐based dietary guidelines|Food and Agriculture Organization of the United Nations. Accessed February 16, 2024. https://www.fao.org/nutrition/education/food-dietary-guidelines/home/en/

[28] Great Britain . (Ed). Dietary Reference Values for Food Energy and Nutrients for the United Kingdom: Report. 18. Impression. TSO; 2008.

[29] Institute of Medicine (U.S.) , Institute of Medicine (U.S.) , Institute of Medicine (U.S.) . (Eds). Dietary Reference Intakes: Applications in Dietary Planning. National Academies Press; 2003. 25057648

[30] National Health and Medical Research Council . Ministry of Health . Nutrient Reference Values for Australia and New Zealand: Including Recommended Dietary Intakes. 2006.

[31] World Health Organization . 2020. Who guidelines on physical activity and sedentary behaviour. Accessed August 9, 2022. https://www.who.int/publications-detail-redirect/9789240015128 33369898

[32] World Obesity Federation Global Obesity Observatory . World Obesity Federation Global Obesity Observatory. Published 2022. Accessed September 12, 2022. https://data.worldobesity.org/

[33] Sharma A , Parkar S , Gaur A , Bagri B . Impact of incarceration on nutritional status and oral health among male inmates of Central Jail of Jaipur city, India. Rev Esp Sanid Penit. 2020;22(3):96‐103. doi:10.18176/resp.00018 33300940 PMC7754537

[34] Rueda ZV , López L , Vélez LA , et al. High incidence of tuberculosis, low sensitivity of current diagnostic scheme and prolonged culture positivity in four Colombian prisons: a cohort study. PLoS One. 2013;8(11):e80592. doi:10.1371/journal.pone.0080592 24278293 PMC3836852

[35] Kayomo MK , Hasker E , Aloni M , et al. Outbreak of tuberculosis and multidrug‐resistant tuberculosis, Mbuji‐Mayi Central Prison, Democratic Republic of the Congo. Emerg Infect Dis. 2018;24(11):2029‐2035. doi:10.3201/eid2411.180769 30334730 PMC6199999

[36] Maruschak, LM . Medical problems of state and federal prisoners and jail inmates, 2011‐12. Published online 2015:23.

[37] Mukhtar S , Khatoon F . Prevalence of risk factors of non‐communicable diseases amongst female prisoners of Pakistan. IOSR J Pharm Biol Sci. 2021;5(2):43‐48.

[38] Rocca D . Prevalence of overweight and obesity in an Italian prison and relation with average term of detention: a pilot study. Ann Ig Med Prev Comm. 2018;(1):51‐56. doi:10.7416/ai.2018.2195 29215131

[39] Argüello‐González AJ , García‐Zazueta MA . Prevalence of overweight and obesity in a Mexican prison. Rev Esp Sanid Penit. 2020;22(2):58‐65. doi:10.18176/resp.00011 32697275 PMC7537363

[40] Shaw NS , Rutherdale M , Kenny J . Eating more and enjoying it less. Women Health. 1985;10(1):39‐57. doi:10.1300/J013v10n01_04 3984358

[41] Wondimu W , Girma B , Sinaga M , Taye A . Undernutrition and associated factors among incarcerated people in Mizan prison institute, southwest Ethiopia. PLoS One. 2021;16(5):e0251364. doi:10.1371/journal.pone.0251364 33974638 PMC8112703

[42] Gould C , Tousignant B , Brian G , et al. Cross‐sectional dietary deficiencies among a prison population in Papua New Guinea. BMC Int Health Hum Rights. 2013;13(1):21. doi:10.1186/1472-698X-13-21 23601963 PMC3637570

[43] Jimoh A , Sabir A . Non communicable diseases among prison inmates in North Western Nigeria. Orient J Med. 2015;27:105‐108.

[44] Clarke JG , Waring ME . Overweight, obesity, and weight change among incarcerated women. J Correct Health Care. 2012;18(4):285‐292. doi:10.1177/1078345812456010 22899812

[45] Lai SW , Liao KF , Chang WL . Assessment of health status among incarcerated men. Am J Med Sci. 2008;335(6):465‐468. doi:10.1097/MAJ.0b013e31815b9d40 18552577

[46] Abera SF , Adane K . One‐fourth of the prisoners are underweight in Northern Ethiopia: a cross‐sectional study. BMC Public Health. 2017;17(1):449. doi:10.1186/s12889-017-4410-9 28506311 PMC5433041

[47] Banu S , Hossain A , Uddin MKM , et al. Pulmonary tuberculosis and drug resistance in Dhaka Central Jail, the largest prison in Bangladesh. PLoS One. 2010;5(5):e10759. doi:10.1371/journal.pone.0010759 20505826 PMC2874010

[48] Bautista‐Arredondo S , González A , Servan‐Mori E , et al. A cross‐sectional study of prisoners in Mexico City comparing prevalence of transmissible infections and chronic diseases with that in the general population. PLoS ONE. 2015;10(7):e0131718. doi:10.1371/journal.pone.0131718 26192811 PMC4508056

[49] Beck, A , Berzofsky, M , Caspar, R , Krebs, C . Sexual victimization in prisons and jails reported by inmates, 2011–12. The Bureau of Justice Statistics is the statistics agency of the U.S. Department of Justice; 2013. Accessed August 22, 2022. https://nicic.gov/resources/nic-library/all-library-items/sexual-victimization-prisons-and-jails-reported-inmates-0

[50] Befus M , Lowy FD , Miko BA , Mukherjee DV , Herzig CTA , Larson EL . Obesity as a determinant of Staphylococcus aureus colonization among inmates in maximum‐security prisons in New York state. Am J Epidemiol. 2015;182(6):494‐502. doi:10.1093/aje/kwv062 26292691 PMC4564937

[51] Binswanger IA , Krueger PM , Steiner JF . Prevalence of chronic medical conditions among jail and prison inmates in the USA compared with the general population. J Epidemiol Community Health. 2009;63(11):912‐919. doi:10.1136/jech.2009.090662 19648129

[52] Brewer‐Smyth K . Obesity, traumatic brain injury, childhood abuse, and suicide attempts in females at risk. Rehabil Nurs. 2014;39(4):183‐191. doi:10.1002/rnj.150 24668743 PMC4227609

[53] Brewer‐Smyth K , Cornelius M , Pohlig RT . Childhood adversity and mental health correlates of obesity in a population at risk. J Correct Health Care. 2016;22(4):367‐382. doi:10.1177/1078345816670161 27742859 PMC5504522

[54] Camplain R , Lininger MR , Baldwin JA , Trotter RT . Cardiovascular risk factors among individuals incarcerated in an Arizona county jail. Int J Environ Res Public Health. 2021;18(13):7007. doi:10.3390/ijerph18137007 34208981 PMC8297210

[55] Choudhry K , Armstrong D , Dregan A . Obesity and weight change in two United Kingdom male prisons. J Correct Health Care. 2019;25(4):328‐337. doi:10.1177/1078345819879925 31722583

[56] Diendéré EA , Traoré K , Bernatas JJ , et al. Prison health priorities in Burkina Faso: a cross‐sectional study in the two largest detention environments in Burkina Faso. Int J Prison Health. 2021;18(1):97‐113. doi:10.1108/IJPH-04-2021-0036 34392661

[57] Drach LL , Maher JE , Braun MJF , Murray SL , Sazie E . Substance use, disordered eating, and weight gain. J Correct Health Care. 2016;22(2):139‐145. doi:10.1177/1078345816634692 26984137

[58] Edwards JSA , Edwards A , Reeve WG . The nutritional content of male prisoners' diet in the UK. Food Serv Technol. 2001;1(1):25‐33. doi:10.1046/j.1471-5740.2001.00003.x

[59] Edwards J , Hartwell H , Reeve W , Schafheitle J . The diet of prisoners in England. Br Food J. 2007;109:216‐232. doi:10.1108/00070700710732547 3

[60] Ehteshamfar SM , Shapouri‐Moghaddam A , Safarian M , Nematy M , Bahrami‐Taghanaki H , Azizi H . Serum selenium concentration in Mashhad prisoners, Iran. Saudi Med J. 2012;33(8):859‐862. 22886118

[61] Hachbardt NB , Hattori TY , do Nascimento VF , et al. Cardiovascular risk in women deprived of freedom from a public prison in Mato Grosso, Brazil. High Blood Press Cardiovasc Prev. 2020;27(2):139‐150. doi:10.1007/s40292-020-00365-2 32144728

[62] Himwaaba G. Incidence and prevalence of hypertension among inmates in three correctional facilities in Lusaka province, Zambia TEXILA Int J PUBLIC Health 2021 9 4 144 https://www.academia.edu/68485399/Incidence_and_Prevalence_of_Hypertension_among_Inmates_in_Three_Correctional_Facilities_in_Lusaka_Province_Zambia 162. 10.21522/TIJPH.2013.09.04.Art013

[63] Jacobs ET , Mullany CJ . Vitamin D deficiency and inadequacy in a correctional population. Nutr Burbank Los Angel Cty Calif. 2015;31(5):659‐663. doi:10.1016/j.nut.2014.10.010 25837209

[64] Johnson C , Chaput JP , Diasparra M , Richard C , Dubois L . Influence of physical activity, screen time and sleep on inmates' body weight during incarceration in Canadian federal penitentiaries: a retrospective cohort study. Can J Public Health. 2019;110(2):198‐209. doi:10.17269/s41997-018-0165-z 30610565 PMC6964490

[65] Kalonji MPG , Coninck GD , Ngongo LO , Ilunga FI , Albert A , Giet D . Nutritional status of inmates in the central prison of Mbuji‐Mayi, Democratic Republic of Congo. Int J Nutr. 2021;6(4):11‐20. doi:10.14302/issn.2379-7835.ijn-21-3926

[66] Khavjou OA , Clarke J , Hofeldt RM , et al. A captive audience: bringing the wisewoman program to South Dakota prisoners. Womens Health Issues. 2007;17(4):193‐201. doi:10.1016/j.whi.2007.02.008 17572105

[67] Khodabakhshifard A , Safarian M , Rostami S , et al. Evaluation of the nutritional status using the anthropometric indices and dietary intakes in the central prison of Mashhad. J Biol Todays World. 2014;3(12):266‐270. doi:10.15412/J.JBTW.01031203

[68] Kosendiak A , Stanikowski P , Domagała D , Gustaw W , Bronkowska M . Dietary habits, diet quality, nutrition knowledge, and associations with physical activity in polish prisoners: a pilot study. Int J Environ Res Public Health. 2022;19(3):1422. doi:10.3390/ijerph19031422 35162445 PMC8834933

[69] Lalem A , Elwafa A , Nouh A . Study of obesity and the rate of cholesterol and triglyceride concentrations among male prison inmates in southern Libya. Nat Sci. 2015;13(1):18‐20.

[70] LaMonaca K , Desai M , May JP , Lyon E , Altice FL . Prisoner health status at three rural Haitian prisons. Int J Prison Health. 2018;14(3):197‐209. doi:10.1108/IJPH-02-2017-0010 30274560

[71] Ekmekcioglu C , Devletlian S , Blasche G , Kundi M . Is there an association between the body mass index and interpersonal violent behavior? J Forensic Sci. 2015;60(5):1350‐1354. doi:10.1111/1556-4029.12790 25946054

[72] Leigey ME , Johnston ME . The prevalence of overweight and obesity among aging female inmates. J Correct Health Care. 2015;21(3):276‐285. doi:10.1177/1078345815588171 26084949

[73] Martínez‐Vicente JR , Baile JI , González‐Calderón MJ . Study of the prevalence of overweight and obesity in a Spanish prison. Nutr Hosp. 2014;30(6):1237‐1239. doi:10.3305/nh.2014.30.6.7782 25433103

[74] Murad R , Qadir M , Qadir A . Prisoners in Karachi: a health and nutritional perspective. Ann Abbasi Shaheed Hosp Karachi Med Dent Coll. 2014;19:67‐72.

[75] Nara K , Igarashi M . Relationship of prison life style to blood pressure, serum lipids and obesity in women prisoners in Japan. Ind Health. 1998;36(1):1‐7. doi:10.2486/indhealth.36.1 9473851

[76] Nessier M , Gerlero SS . Nutritional status behind bars: descriptive study of the nutritional status of a group of women deprived of liberty in a detention criminal unit in the city of Santa Fe (Argentina). Rev Esp Nutr Comun. 2012;18:91‐97.

[77] Noeske J , Kuaban C , Amougou G , Piubello A , Pouillot R . Pulmonary tuberculosis in the central prison of Douala, Cameroon. East Afr Med J. 2006;83(1):25‐30. doi:10.4314/eamj.v83i1.9357 16642747

[78] Nwosu BU , Maranda L , Berry R , et al. The vitamin D status of prison inmates. PLoS ONE. 2014;9(3):e90623. doi:10.1371/journal.pone.0090623 24598840 PMC3944727

[79] Oyedokun, T , Onabanjo, O , Oyedokun, TJ . Dietary pattern, nutritional and health status of inmates in Ibara prison, Abeokuta, Ogun state, Nigeria. Published online January 1, 2018:26‐32. 10.5829/idosi.ajn.2018.26.32

[80] Rahfiludin M , Pangestuti D , Fatimah S , Suroto . The impact of counseling on the improvement of nutritional knowledge and physical activities on women prisoners (a study at women penitentiary institution class II a Semarang). Indian J Public Health Res Dev. 2019;10:958. doi:10.5958/0976-5506.2019.00626.0

[81] Ravaoarisoa L , Pharlin AH , Andriamifidison NZR , et al. Nutritional status of female prisoners in Antanimora prison, Madagascar. Pan Afr Med J. 2019;33:119. doi:10.11604/pamj.2019.33.119.18170 31489097 PMC6711675

[82] Ross J , Field C , Kaye S , Bowman J . Prevalence and correlates of low self‐reported physical health status among prisoners in New South Wales, Australia. Int J Prison Health. 2019;15(2):192‐206. doi:10.1108/IJPH-06-2018-0039 31172857

[83] Simeni Njonnou SR , Boombhi J , Etoa Etoga MC , et al. Prevalence of diabetes and associated risk factors among a group of prisoners in the Yaoundé Central Prison. J Diabetes Res. 2020;2020:e5016327. doi:10.1155/2020/5016327 PMC7003275 32047824

[84] Sioen I , Hacquebard M , Hick G , et al. Effect of ALA‐enriched food supply on cardiovascular risk factors in males. Lipids. 2009;44(7):603‐611. doi:10.1007/s11745-009-3307-5 19452183

[85] Togas C Raikou M Niakas D An assessment of health‐related quality of life in a male prison population in Greece: associations with health‐related characteristics and characteristics of detention BioMed Res Int 2014 2014. doi:10.1155/2014/274804, 2014, 1, 274804 25093161 PMC4100399

[86] Vera‐Remartínez EJ , Borraz‐Fernández JR , Domínguez‐Zamorano JA , et al. Prevalence of chronic diseases and risk factors among the Spanish prison population. Rev Esp Sanid Penit 2014;16(2):38–47. Accessed June 9, 2022. https://scielo.isciii.es/scielo.php?script=sci_abstract&pid=S1575-06202014000200003&lng=en&nrm=iso&tlng=en, doi:10.4321/S1575-06202014000200003 25072788

[87] Voller F , Silvestri C , Martino G , et al. Health conditions of inmates in Italy. BMC Public Health. 2016;16(1):1162. doi:10.1186/s12889-016-3830-2 27852256 PMC5112742

[88] Winetsky DE , Almukhamedov O , Pulatov D , Vezhnina N , Dooronbekova A , Zhussupov B . Prevalence, risk factors and social context of active pulmonary tuberculosis among prison inmates in Tajikistan Mokrousov I, ed. PLoS One. 2014;9(1):e86046. doi:10.1371/journal.pone.0086046 24465861 PMC3896449

[89] Wright NM , Hearty P , Allgar V . Prison primary care and non‐communicable diseases: a data‐linkage survey of prevalence and associated risk factors. BJGP Open. 2019;3(2):bjgpopen19X101643. doi:10.3399/bjgpopen19X101643 PMC6662881 31366674

[90] Wolff N , Shi J , Fabrikant N , Schumann BE . Obesity and weight‐related medical problems of incarcerated persons with and without mental disorders. J Correct Health Care. 2012;18(3):219‐232. doi:10.1177/1078345812445270 22569903

[91] Young M , Waters B , Falconer T , O'Rourke P . Opportunities for health promotion in the Queensland women's prison system. Aust N Z J Public Health. 2005;29(4):324‐327. doi:10.1111/j.1467-842X.2005.tb00201.x 16222928

[92] Maddan S , Walker JT , Miller JM . Does size really matter? Soc Sci J. 2008;45(2):330‐344. doi:10.1016/j.soscij.2008.03.009

[93] Martínez‐Delgado MM , Ramírez‐López C . Cardiovascular health education intervention in the prison of Soria. Rev Esp Sanid Penit. 2016;18(1):5‐11. doi:10.4321/S1575-06202016000100002 26997287

[94] Ullah MH , Fawad DA , Saqib DM , Gul DAM , Jan DHU . Frequency of cardiovascular risk factors among prisoners. Pak Heart J. 2010;43(1‐2). doi:10.47144/phj.v43i1-2.110

[95] Saleh ZT , Connell A , Lennie TA , et al. Cardiovascular disease risk predicts health perception in prison inmates. Clin Nurs Res. 2019;28(2):235‐251. doi:10.1177/1054773817740534 29117723

[96] Packham C , Butcher E , Williams M , Miksza J , Morriss R , Khunti K . Cardiovascular risk profiles and the uptake of the NHS Healthcheck programme in male prisoners in six UK prisons: an observational cross‐sectional survey. BMJ Open. 2020;10(5):e033498. doi:10.1136/bmjopen-2019-033498 PMC7253003 32448789

[97] Gebremariam MK , Nianogo RA , Arah OA . Weight gain during incarceration: systematic review and meta‐analysis. Obes Rev. 2018;19(1):98‐110. doi:10.1111/obr.12622 29024549

[98] Agyapong NAF , Annan RA , Apprey C . Prevalence of risk factors of cardiovascular diseases among prisoners: a systematic review. Nutr Food Sci. 2017;47(6):896‐906. doi:10.1108/NFS-06-2017-0114

[99] World Health Organization . 2017. The double burden of malnutrition: policy brief; Accessed August 22, 2022. https://www.who.int/publications-detail-redirect/WHO-NMH-NHD-17.3

[100] Dolan K , Wirtz AL , Moazen B , et al. Global burden of HIV, viral hepatitis, and tuberculosis in prisoners and detainees. Lancet Lond Engl. 2016;388(10049):1089‐1102. doi:10.1016/S0140-6736(16)30466-4 27427453

[101] World Health Organisation . Nutrition. Published February 22, 2023. Accessed March 8, 2023. https://www.who.int/health-topics/nutrition

[102] FAO F and AO of the UN . Dietary guidelines and sustainability. Food and Agriculture Organization of the United Nations. Published 2022. Accessed January 27, 2022. http://www.fao.org/nutrition/education/food-dietary-guidelines/background/sustainable-dietary-guidelines/en/

[103] Agyapong NA , Annan R , Apprey C . Assessment of food and nutrient provision within prisons in the Ashanti region of Ghana. Asian Food Sci J. 2018;4:1‐6. doi:10.9734/AFSJ/2018/43579

[104] Collins SA , Thompson SH . What are we feeding our inmates? J Correct Health Care. 2012;18(3):210‐218. doi:10.1177/1078345812444875 22553283

[105] Cook EA , Lee YM , White BD , Gropper SS . The diet of inmates. J Correct Health Care. 2015;21(4):390‐399. doi:10.1177/1078345815600160 26276135

[106] Korir, JC . Evaluating the nature of food served in selected prisons in Kenya. Afr J Hosp Tour Leis 2017;6(2):18. http://www.ajhtl.com/uploads/7/1/6/3/7163688/article_12_vol_6__2__2017.pdf

[107] Johnson C , Chaput JP , Diasparra M , Richard C , Dubois L . Canadian federal penitentiaries as obesogenic environments: a retrospective cohort study. CMAJ Open. 2018;6(3):E347‐E352. doi:10.9778/cmajo.20180044 PMC6182125 30154218

[108] WHO . Healthy diet. Published April 29, 2020. Accessed January 25, 2022. https://www.who.int/news-room/fact-sheets/detail/healthy-diet

[109] Stanikowski P , Michalak‐Majewska M , Domagała D , Jabłońska‐Ryś E , Sławińska A . Implementation of dietary reference intake standards in prison menus in Poland. Nutrients. 2020;12(3):E728. doi:10.3390/nu12030728 PMC7146611 32164205

[110] Kavithe KR , Dorcus MDK , Maoga WN . Dietary intake and factors affecting food service of male prisoners living with human immunodeficiency virus at selected prisons in Kenya. Int J Nutr Metab. 2018;10(2):6‐15. doi:10.5897/IJNAM2017.0229

[111] Zalwango C , Ayebare P , Mwanja P , et al. Prevalence and factors associated with ocular morbidity among prisoners of Luzira prison (Uganda). BMC Ophthalmol. 2021;21(1):278. doi:10.1186/s12886-021-02035-w 34261442 PMC8278745

[112] Amogne W , Nimani M , Shemsedin I , et al. An epidemic of scurvy, identified based on lower extremity swelling, in a southern Ethiopian prison. Am J Trop Med Hyg. 2021;105(2):511‐516. doi:10.4269/ajtmh.20-1246 34181570 PMC8437177

[113] Bennett M , Coninx R . The mystery of the wooden leg: vitamin C deficiency in East African prisons. Trop Doct. 2005;35(2):81‐84. doi:10.1258/0049475054036896 15970026

[114] Mathenge W , Kuper H , Myatt M , Foster A , Gilbert C . Vitamin A deficiency in a Kenyan prison. Trop Med Int Health. 2007;12(2):269‐273. doi:10.1111/j.1365-3156.2006.01780.x 17300635

[115] Papa V , Tafuri D , Vaccarezza M . Could physical activity have any role in cardiovascular disease prevention in prisoners? A systematic review. Int J Environ Res Public Health. 2021;18(5):2307. doi:10.3390/ijerph18052307 33652816 PMC7956477

[116] Mannocci A , Mipatrini D , D'Egidio V , et al. Health‐related quality of life and physical activity in prison: a multicenter observational study in Italy. Eur J Public Health. 2018;28(3):570‐576. doi:10.1093/eurpub/ckx183 29069337

