# Supplementary material for: Prevalence of overweight and obesity in incarcerated individuals in developed and developing countries: A systematic review and meta‐analysis
Source: Obes Rev. 2025 Feb 12;26(6):e13906. doi: 10.1111/obr.13906 (PMC12069163; doi:10.1111/obr.13906)
Supplement: Supplementary file 1 — Table S1. Search strategy. Table S2. Risk of Bias Critical Appraisal. [file OBR-26-e13906-s001.pdf]

Prevalence of overweight and obesity in incarcerated individuals in developed and developing countries: a systematic review and meta-analysis.

Leonida Nyarwaba Mosomi, Magaly Aceves-Martins, Alexandra M. Johnstone, Baukje de Roos

The Rowett Institute, University of Aberdeen, AB25 2ZD, United Kingdom

E-mail: b.deroos@abdn.ac.uk

Supporting Information

Table 1. Search strategy

#	**Query**
1	exp Prisoners/ or exp Prisons/
2	(felon* or jail* or custod* or offender* or inmate* or incarcerat* or gaol* or officer*).ti,ab.
3	1 or 2
4	(nutrition* or nutritional status or body mass or obes* or overweigh or underweigh or body weight).ti,ab.
5	3 and 4
6	limit 5 to humans
7	limit 6 to (" young adult (19 to 24 years)" or "adult (19 to 44 years)" or "young adult and adult (19-24 and 19-44)" or "middle age (45 to 64 years)" or "middle aged (45 plus years)") [Limit not valid in Embase; records were retained]
8	limit 7 to adult <18 to 64 years> [Limit not valid in Ovid MEDLINE(R),Ovid MEDLINE(R) Daily Update,Ovid MEDLINE(R) PubMed not MEDLINE,Ovid MEDLINE(R) In-Process,Ovid MEDLINE(R) Publisher; records were retained]
9	limit 8 to (dutch or english or portuguese or spanish)
10	remove duplicates from 9

Table 2. Risk of Bias Critical Appraisal

**STUDY ID**<br>Country	Were the criteria for inclusion in the sample clearly defined?	Were the study subjects and the setting described in detail?	Was the exposure measured in a valid and reliable way?	Were objective, standard criteria used for measurement of the condition?	Were confounding factors identified?	Were strategies to deal with confounding factors stated?	Were the outcomes measured in a valid and reliable way?	Was appropriate statistical analysis used?	Overall appraisal total score out of 8
**High-Income Countries**									
**Al-Rousan 2017**[10]<br>United States	Yes	Yes	Yes	Yes	Yes	No	Yes	Yes	7/8<br>Good
**Johnson 2018**[11,64]<br>Canada	Yes	Yes	Yes	Yes	Yes	Yes	Yes	Yes	8/8<br>Good
**Lagarrigue 2017**[12]<br>France	Yes	Yes	Yes	Yes	Yes	No	Yes	Yes	7/8<br>Good
**Gates 2015**[13]<br>United States	Yes	Yes	Yes	Yes	Yes	No	Yes	Yes	7/8<br>Good
**D'Souza 2005**[15]<br>Australia	Yes	Yes	Yes	Yes	Yes	Yes	Yes	Yes	8/8<br>Good
**Gray 2021**[16]<br>United Kingdom	Yes	Yes	Yes	Yes	Yes	No	Yes	Yes	7/8<br>Good
**Hannan-Jones 2016**[17]<br>Australia	Yes	Yes	Yes	Yes	Yes	No	Yes	Yes	7/8<br>Good
**Nucci 2020**[19]<br>Italy	Yes	Yes	Yes	Yes	Yes	No	Yes	Yes	7/8<br>Good
**Plugge 2009**[25]<br>United Kingdom	Yes	Yes	Yes	Yes	Yes	No	Yes	Yes	7/8<br>Good
**Shaw 1985**[40]<br>United States	Yes	Yes	Yes	Yes	Yes	No	Yes	Yes	7/8<br>Good
**Maruschak 2015**[36,49]<br>United States	Yes	Yes	Yes	Yes	Yes	No	Yes	Yes	7/8<br>Good
**Rocca 2018**[38]<br>Italy	Yes	Yes	Yes	Yes	Yes	No	Yes	Yes	7/8<br>Good
**Clarke 2012**[44]<br>United States	Yes	Yes	Yes	Yes	Yes	No	Yes	Yes	7/8<br>Good
**Lai 2008**[45]	Yes	Yes	Yes	Yes	Yes	No	Yes	Yes	7/8

Taiwan									Good
**Befus 2015**[50]<br>United States	Yes	Yes	Yes	Yes	Yes	Yes	Yes	Yes	7/8<br>Good
**Binswanger 2009**[51]<br>United States	Yes	Yes	Unclear	Yes	Yes	No	Yes	Yes	6/8<br>Moderate
**Brewer-Smyth 2014**[52]<br>United States	Yes	Yes	Unclear	Yes	Yes	No	Unclear	Yes	5/6<br>Moderate
**Brewer-Smyth 2016**[53]<br>United States	Yes	Unclear	Unclear	Yes	Yes	No	Unclear	Yes	4/8<br>Poor
**Camplain 2021**[54]<br>United States	Yes	Yes	Unclear	unclear	Yes	No	No	Yes	4/8<br>Poor
**Choudhry 2019**[55]<br>United Kingdom	Yes	Yes	Unclear	Yes	Yes	No	Unclear	Yes	5/8<br>Moderate
**Drach 2016**[57]<br>United States	Yes	Yes	Yes	Yes	Yes	No	Yes	Yes	7/8<br>Good
**Edwards 2001**[58,59]<br>United Kingdom	Yes	Yes	Yes	Yes	Yes	No	Unclear	Yes	6/8<br>Moderate
**Hachbardt 2020**[61]<br>Italy	Yes	Yes	Yes	Yes	Yes	Yes	Yes	Yes	8/8<br>Good
**Jacobs 2015**[63]<br>United States	Yes	Yes	Yes	Yes	Yes	No	Yes	Yes	7/8<br>Good
**Khavjou 2007**[66]<br>United States	Yes	Yes	Yes	Yes	Yes	No	Yes	Yes	7/8<br>Good
**Kosendiak 2022**[68]<br>Poland	Yes	Yes	Yes	Yes	Yes	No	Yes	Yes	7/8<br>Good
**Ekmekcioglu 2015**[71]<br>Austria	Yes	Yes	Yes	Yes	Yes	No	Yes	Yes	7/8<br>Good
**Leigey 2015**[72]<br>United States	Yes	Yes	Yes	Yes	Yes	No	Yes	Yes	7/8<br>Good
**Martínez-Vicente 2014**[73]<br>Spain	No	No	Yes	Unclear	Yes	No	Yes	Yes	4/8<br>Poor
**Nara 1998**[75]<br>Japan	Yes	Yes	Yes	Yes	Yes	No	unclear	yes	6/8<br>Moderate
**Nwosu 2014**[78]<br>United States	yes	yes	yes	yes	Yes	No	yes	yes	7/8<br>Good
**Ross 2019**[82]<br>Australia	Yes	Yes	No	Yes	Yes	No	Unclear	yes	6/8<br>Moderate
**Sioen 2009**[84]	Yes	Yes	Yes	Yes	Yes	No	Yes	Yes	7/8

Belgium									Good
**Togas 2014**[85]<br>Greece	Yes	Yes	Yes	Yes	Yes	Yes	Yes	Yes	8/8<br>Good
**Vera-Remartínez 2014**[86]<br>Spain	Yes	Yes	Yes	Yes	Yes	No	Yes	Yes	8/8<br>Good
**Voller 2016**[87]<br>Italy	Yes	Yes	Yes	Yes	Yes	Yes	Yes	Yes	8/8<br>Good
**Wright, 2019**[89]<br>United Kingdom	Yes	Yes	Yes	Yes	Yes	No	Yes	Yes	7/8<br>Good
**Wolff 2012** [90]<br>United States	Yes	Yes	Yes	Yes	Yes	No	Yes	Yes	7/8<br>Good
**Young 2005**[91]<br>Australia	Yes	Yes	Yes	Yes	Yes	No	Yes	Yes	7/8<br>Good
**Maddan 2008**[92]<br>United States	Yes	Yes	Yes	Yes	Yes	No	Yes	Yes	7/8<br>Good
**Martínez-Delgado 2016**[93]<br>Spain	Yes	Yes	Yes	Yes	Yes	No	Yes	Yes	7/8<br>Good
**Saleh 2019**[95]<br>United States	Yes	Yes	Yes	Yes	Yes	No	Yes	Yes	7/8<br>Good
**Packham 2020**[96]<br>United Kingdom	Yes	Yes	Yes	Yes	Yes	No	Yes	Yes	7/8<br>Good
**Upper-Middle-Income Countries**									
**Audi 2018**[18]<br>Brazil	Yes	Yes	Yes	Yes	Yes	No	Yes	Yes	7/8<br>Good
**Rueda 2013**[34]<br>Colombia	Yes	Yes	Yes	Yes	Yes	No	Yes	Yes	7/8<br>Good
**Argüello-González 2020**[39]<br>México	Yes	Yes	Yes	Yes	Yes	No	Yes	Yes	7/8<br>Good
**Bautista-Arredondo 2015**[48]<br>Mexico	Yes	Yes	Yes	Yes	Yes	No	Yes	Yes	7/8<br>Good
**Lalem et al., 2015**[69]<br>Libya	Yes	Yes	Yes	Yes	Yes	No	Yes	Yes	7/8<br>Good
**Nessier 2012**[76]<br>Argentina	Yes	Yes	Yes	Yes	Yes	No	Yes	Yes	7/8<br>Good
**Lower-Middle Income Countries**									
**Noeske 2011**[6]<br>Cameroon	Yes	Yes	Yes	Yes	Yes	No	Unclear	yes	6/8<br>Moderate
**Sharma 2020**[33]	Yes	Yes	Yes	Yes	Yes	No	Yes	Yes	7/8

India									Good
**Mukhtar 2013**[37] Pakistan	Yes	Yes	Yes	Yes	Yes	No	Yes	Yes	7/8 Good
**Gould 2013**[42] Papua New Guinea	Yes	Yes	Yes	Yes	Yes	No	Yes	Yes	7/8 Good
**Jimoh 2015**[43] Nigeria	Yes	Yes	Yes	Yes	Yes	No	Yes	Yes	7/8 Good
**Banu 2010**[47] Bangladesh	Yes	Yes	Yes	Yes	Yes	No	Yes	Yes	7/8 Good
**Khodabakhshifard 2014**[60,67] Iran	Yes	Yes	Yes	Yes	Yes	No	Yes	Yes	7/8 Good
**Himwaaba 2021**[62] Zambia	Yes	Yes	Yes	no	Yes	Yes	Yes	Yes	7/8 Good
**LaMonaca 2018**[70] Haiti	Yes	Yes	No	Yes	Yes	No	Yes	Yes	6/8 Moderate
**Murad 2014**[74] Pakistan	Yes	Yes	Yes	Yes	Yes	No	Yes	Yes	7/8 Good
**Noeske 2006** [77] Cameroon	Yes	Yes	Yes	Yes	Yes	No	Unclear	Yes	6/8 Moderate
**Oyedokun 2018**[79] Nigeria	Yes	Yes	Yes	Yes	Yes	No	Yes	Yes	7/8 Good
**Rahfiludin 2019**[80] Indonesia	Yes	Yes	Yes	Yes	Yes	No	Yes	Yes	7/8 Good
**Simeni 2020**[83] Cameroon	Yes	Yes	Yes	Yes	Yes	No	Yes	Yes	7/8 Good
**Winetsky 2014**[88] Tajikistan	Yes	Yes	Yes	Yes	Yes	No	Yes	Yes	7/8 Good
**Ullah 2010**[94] Pakistan	Yes	Yes	Yes	Yes	Yes	No	Yes	Yes	7/8 Good
**Low-Income Countries**									
**Kayomo 2018**[35] Democratic Republic of the Congo	Yes	Yes	Unclear	Yes	Yes	No	Unclear	Yes	5/8 Moderate
**Wondimu 2021**[41] Ethiopia	Yes	Yes	Unclear	Yes	Yes	No	Unclear	Yes	6/8 Moderate
**Abera 2017**[46] Ethiopia	Yes	Yes	Unclear	Yes	Yes	No	Unclear	Yes	5/8 Moderate
**Diendéré 2021**[56] Burkina Faso	Yes	Yes	Unclear	Yes	Yes	No	Unclear	Yes	5/8 Moderate

**Kalonji 2021**[65]<br>Democratic Republic of the Congo	Yes	Yes	Yes	Yes	Yes	No	Unclear	Yes	6/8<br>Moderate
**Ravaoarisoa 2019**[81]<br>Madagascar	Yes	Yes	Unclear	Yes	Yes	No	Unclear	Yes	6/8<br>Moderate
